# Ensuring end-to-end privacy in IoT-based healthcare systems through hyperledger fabric and identity mixer

**DOI:** 10.1371/journal.pone.0343233

**Published:** 2026-03-27

**Authors:** Maryam Nasr Esfahani, Behrouz Shahgholi Ghahfarokhi, Shahram Etemadi Borujeni

**Affiliations:** 1 Department of Electrical and Computer Engineering, Isfahan University of Technology, Isfahan, Iran; 2 Faculty of Computer Engineering, University of Isfahan, Isfahan, Iran; Maulana Abul Kalam Azad University of Technology West Bengal, INDIA

## Abstract

Healthcare information is often sensitive and confidential, and any unauthorized disclosure can jeopardize patient privacy. Centralized approaches currently used to protect patient information are vulnerable to a single point of failure and the risk of data breaches from central servers. Conversely, distributed blockchain-based methods tend to focus only on safeguarding data privacy for certain segments of the communication path from sensors to end users. This leaves a gap in methodologies that ensure both location and data privacy throughout the entire communication from sensors to healthcare data users via blockchain technology. In this paper, we propose using Hyperledger Fabric to ensure the immutability of patient data and to address the single point of failure issue. Additionally, to maintain end-to-end data and location privacy, our proposed scheme integrates the Idemix protocol suit alongside Hyperledger Fabric and employs a lightweight communication protocol for interactions between healthcare sensors and gateways. Compared to previous methods, our approach not only facilitates privacy features such as patient anonymity and untraceability but also guarantees data integrity, confidentiality, mutual authentication, and resilience against a variety of security threats, including collusion among internal entities. While the implementation of blockchain may introduce additional computational overhead for service providers compared to centralized systems, this trade-off is outweighed by the significant enhancements in security as evidenced by our analyses and evaluations.

## Introduction

Due to the increasing costs of current healthcare services and the prevalence of chronic diseases [1], it has been predicted that healthcare systems change from hospital-centered to home-centered in 2030 [1]. Therefore, the need for remote monitoring of patients has led to using the Internet of Things (IoT) concept in healthcare systems. In such systems, bio-medical and context information are gathered through the sensors such as wearable ones and the information is processed at various levels to make prescriptions and possibly drug injections. These systems not only improve the quality of healthcare services but also reduce healthcare costs [2,3].

Continuous patient monitoring through Wireless Body Area Networks (WBAN) increases the early diagnosis of emergency conditions of patients and provides healthcare services for people with different degrees of disability [4]. These networks include a number of sensors on the surface of the body, inside the tissues, or on clothing. They should gather, process, and communicate healthcare information [5]. In these networks, some gateways receive sensor information and send it to remote servers [6,7]. Each sensor usually collects and processes some vital signs [5,8]. Those sensors have small batteries, low storage, and limited communication capabilities [5]. Due to resource constraints of sensors, conventional security protocols are not suitable [9,10] and lightweight solutions are necessary [10–12]. A solution is using smart gateways to collect data from WBANs and providing security services on behalf of the sensors [13–15].

Providing end-to-end data and location privacy of patients is an important issue in healthcare systems [9,11]. Many patients do not like to disclose their illness to others. Another concern of patients is revealing their location to others since their location should only be revealed to emergency centers. Privacy-preserving methods are divided into centralized and distributed ones. The centralized methods for protecting the data and location privacy of the patients (such as [16–23]) face the risk of a single point of failure problem and the possibility of privacy leakage from parts of the system. Privacy leakage is due to the fact that those studies assume some parts of the system as trusted while they may be curious despite following the protocol appropriately. For example, Ref. [2,10,16–18,23] just protect the privacy against external attackers and assume all entities as trusted ones.

Some studies place their trust in gateways or service providers [11,19,22,24], while others consider these gateways and healthcare service providers to be untrustworthy [20,21,25]. Only the study in [25] adopts the perspective that all system entities may be curious. Most of the studies mentioned in [16,17,21,22,26] primarily emphasize data privacy, while a few, such as [27], concentrate solely on location privacy. Some studies [10,11,18,20,24,25,28] address both data and location privacy. Our previous work [25] proposes a centralized scheme to preserve both data and location privacy, operating under the assumption that all system entities are untrusted. However, this approach is vulnerable to a single point of failure and is susceptible to collusion attacks, as noted in [25].

Blockchain (BC) is used as a distributed solution to preserve patients’ privacy in several studies [29–33]. Some others [34–41] utilize BC for access control in healthcare systems. Moreover, some studies [42–45] use BC with attribute-based encryption (ABE) methods to gain security features such as authentication, confidentiality, and integrity of healthcare data. Other studies [46–50] utilize patient-centric agents as well as BC to protect privacy.

However, the majority of earlier studies concentrate solely on safeguarding data privacy for specific segments of the end-to-end communication from sensors to the final users.

An end-to-end privacy-preserving method that maintains location and data privacy using blockchain has been proposed in previous work [51]. However, the approach presented in Ref. [51] is susceptible to several security vulnerabilities and attacks. In the initial stages of the authentication and data transmission algorithms, a modification attack occurs on critical parameters of the authentication process, leading to the misinterpretation of patient information in subsequent stages. This leads to compromising message integrity. Furthermore, message authentication is compromised due to another modification attack, although the end users finally recognize the modified information too late at the final stage, once it has already been entered into the healthcare information database. Additionally, some assumptions made in the aforementioned paper [51] have weakened the proposed solution.

Consequently, there is a notable absence of a comprehensive privacy-preserving approach that ensures both location and data privacy throughout the end-to-end communication from sensors to healthcare data users using blockchain technology. To address these vulnerabilities, we utilize the Hyperledger Fabric blockchain framework in conjunction with the Idemix protocol suite to establish end-to-end privacy in healthcare systems. Leveraging Hyperledger Fabric’s capabilities for immutable and secure data transaction storage, we incorporate this framework into our proposed method. Idemix facilitates anonymous authentication and privacy protection through a blind signature scheme and efficient zero-knowledge proofs. We assume that all system entities are semi-honest, meaning that they adhere to the protocol while remaining curious. The key contributions of our work are outlined as follows:

- As mentioned previously, our earlier work [51] is vulnerable to modification attacks due to weak authentication in gateways. To address this issue highlighted in Ref. [51], we propose using Idemix for authentication. This new scheme aims to preserve the end-to-end privacy and anonymity of patients in a blockchain-based healthcare system, effectively resolving the problems identified in Ref. [51]. The proposed scheme safeguards patients’ privacy against violations from both external attackers and insider threats.
- A secure protocol is proposed to present the mentioned security features with the help of BC and Idemix.
- The proposed scheme provides both data privacy and location privacy of the patients, in contrast to most previous BC-based methods that are only preserving healthcare data’s privacy.
- A lightweight protocol is used to anonymously authenticate the sensor to the gateway and maintain its data privacy using light operations such as XOR and hash. Then, Idemix services are employed by the gateway to complete the procedure on behalf of the sensor.

This paper is organized as follows. First, we review recent literature on methods for protecting patient privacy. Next, we provide some preliminary background information before detailing the proposed scheme. We then cover the security analysis and performance evaluations. The paper concludes with a summary of our work and final remarks.

## Related work

Numerous studies have applied blockchain (BC) to protect patient privacy in IoT-based healthcare environments [38,41,48,49,52–62]. Representative contributions include the two-layer blockchain framework S2HS for securing interactions among healthcare entities [55]; a blockchain-based electronic health record system that facilitates access to medical records and hospital resources [56]; and decentralized, privacy-preserving algorithms that enable distributed computation without revealing sensitive data while relying on blockchain for transparent record-keeping [57]. Reviews also document deployed healthcare applications such as MedRec, MeDShare, OmniPHR, Pervasive Social Network (PSN), and Healthcare Data Gateway [29–33,63].

Some approaches retain health records in local databases managed by healthcare providers and use blockchain primarily as an access-control layer, storing only hashed pointers and access policies on distributed ledgers to improve scalability, integrity, and availability [38,41]. Ref. [41] additionally proposes a patient-centric management platform that supports privacy-preserving storage. Other works concentrate explicitly on data confidentiality by encrypting information and storing it in distributed file systems such as IPFS [58], or by applying lightweight obfuscation techniques (e.g., interleaving encoders) to conceal raw data semantics [59].

Several efforts address resource constraints at sensing nodes. For example, lightweight security protocols, cluster-head-based aggregation, and ring signatures have been used to reduce sensor-side computation and to anonymize miners that verify data blocks prior to blockchain publication on cloud servers [62]. Similarly, Wang et al. propose an e-health architecture tailored for resource-constrained hardware in which WBAN gateways aggregate data and forward it to the blockchain over secure channels [61].

**Fig 1:**
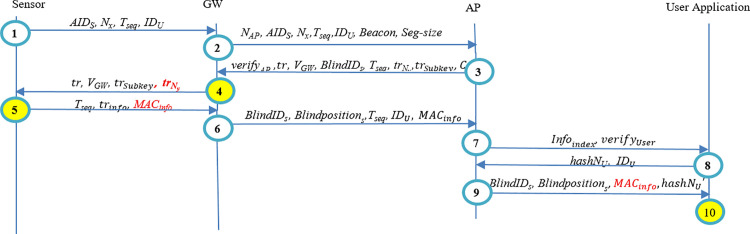
Modification attack in the Forth step of in the authentication protocol of the method of Ref. [51].

**Fig 2:**
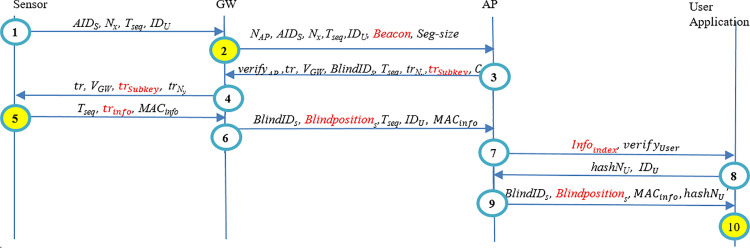
Modification attack in the second step of the authentication protocol of Ref. [51].

Authors’ prior work [51] presents a three-layer blockchain architecture employing zero-knowledge proofs and ring signatures to protect end-to-end data and location privacy, with computation of sensors being offloaded to gateways and access points. The protocol comprises entity registration, mutual authentication, information transfer, and information-access. However, there are vulnerabilities in that protocol. Modification attacks on sensitive early-stage parameters (e.g., ${tr}_{N_{y}}$ or *Beacon*) can cause incorrect ${MAC}_{info}$ or *Info* values to be produced and recorded. Although the end user detects integrity or authenticity violations at the final verification step, erroneous data is stored in the provider’s database (see Figs 1 and 2). Fig 1 schematically depicts the authentication and information transmission algorithm outlined in Ref. [51]. This representation omits detailed per-step operations by each entity, focusing instead on sensitive parameters such as ${tr}_{N_{y}}$ and ${MAC}_{info}$. If an attacker alters the value of ${tr}_{N_{y}}$, then the sensor will incorrectly compute ${MAC}_{info}$ in Step 5. This ${MAC}_{info}$ parameter is subsequently used in Step 10 to verify the integrity and accuracy of the patient's information. Consequently, the end user fails to provide early validation, i.e., detects the mismatch in ${MAC}_{info}$ at the final verification step (Step 10).

Moreover, another modification undermines message authenticity, leading to a situation where end users only become aware of the incorrect information at the final stage, after it has already been recorded in the healthcare information database. Similar to Fig 1, Fig 2 illustrates a modification attack on the authentication and information transfer algorithm described in [51]. This figure specifically highlights sensitive parameters, such as *Beacon*. If an attacker modifies the *Beacon* parameter in Step 2, it causes subsequent entities to incorrectly compute several critical parameters across Steps 3–10. Among these, the *Info* parameter, containing the patient's healthcare information, is paramount. The *Info* is ultimately delivered to the end user via ${Blindposition}_{s}$ *i*n Step 10. Consequently, the end user detects the compromised information too late, i.e., in the final step (Step 10), while the incorrect information has already been recorded in the database by the Application Provider (AP) in Step 7.

While frameworks such as PREHEALTH [64] and other Fabric/Idemix-based solutions leverage Hyperledger Fabric and identity-mixing techniques to provide anonymity and prevent traceability, they often neglect practical concerns such as latency or do not cover the entire communication path from sensors to end users [64–67]. In summary, existing blockchain-based solutions commonly protect portions of the communication chain, but simultaneous end-to-end protection of both patient’s data and location remains inadequately addressed.

## Background

### IoT-based healthcare systems architecture

IoT-based healthcare architectures commonly comprise three domains: (1) sensors and actuators that are typically lightweight devices which collect physiological and contextual data and communicate with gateways (patient mobile devices or public gateways in clinics/hospitals); (2) communication infrastructure, which consists of edge gateways that support WBAN protocols and mediate between sensors and backend services; and (3) healthcare service providers consisting applications and databases that receive sensor data and present it to end users (e.g., doctors).

### Hyperledger fabric

Blockchain can preserve identity privacy (e.g., using distinct public keys per transaction to prevent tracking) [68–70]. Blockchains are categorized as public, private (permissioned), or consortium, with private/permissioned platforms often preferred for sensitive data [57,71,72]. We adopt Hyperledger Fabric for its privacy, modularity, and lower consensus cost relative to public platforms. Key Fabric concepts relevant to healthcare systems are as follows:

- Channels: private subnets with separate ledgers for confidential communication among authorized members [73].
- Nodes: clients (submit transactions), peers (endorse/commit transactions), and ordering nodes (order transactions into blocks) [74,75]. Peers may assume roles such as endorsing, committing, leader, or anchor.
- Fabric CA and MSP: Fabric CA issues certificates; the Membership Service Provider (MSP) abstracts membership, certificate validation, and authentication, defining trusted CAs and domain roles [76].
- Ledger and world state: the ledger comprises an immutable blockchain plus a world state (key–value store via LevelDB or CouchDB) that reflects current state for efficient queries [77].
- Chaincode: smart contracts executed in containerized environments whose transactions are recorded in the ledger [78].

Transaction lifecycle in Hyperledger Fabric includes: (1) proposal and endorsement where client sends signed proposal and peers execute chaincode and endorse; (2) submission and ordering where client submits signed envelope to ordering service which creates blocks; and (3) validation and commitment where peers validate endorsements and read–write sets, commit blocks to the ledger, and emit commit events [79,80].

### IDEMIX

**Fig 3:**
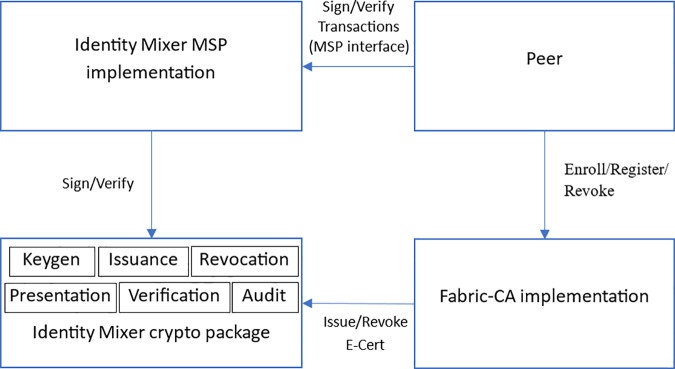
Identity Mixer implementation for Hyperledger Fabric [80].

Idemix provides cryptographic identity privacy (untraceability and selective disclosure) via blind signatures and zero-knowledge proofs [80,81]. Roles include issuer, user, and prover. Versions 1.2 and later of Fabric support Idemix, where Fabric CA issues Idemix credentials, the Fabric Java SDK acts as the user API, and the Idemix MSP verifies presentations [64,80]. Fabric’s Idemix implementation includes CA services, an Idemix MSP, and core cryptographic packages for key/credential issuance and presentation token generation (Fig 3).

- *CA Service: It utilizes the Identity Mixer crypto package to issue ECert credentials.*
- *Idemix MSP implementation: it signs and verifies the transactions with the help of the Identity Mixer crypto package* [80]*.*
- *Core Identity Mixer crypto package: it creates the keys of the issuer, issues credentials, and generates and verifies presentation tokens.*

In Hyperledger Fabric, certificate revocation is managed using Certificate Revocation Information (CRI). The issuer (Fabric CA) revokes credentials, and this revocation is recorded in the CRI [82]. The client (prover) uses the CRI to generate a non-revocation proof, which is verified by the Idemix MSP to ensure the credential is not revoked. The Gateway (GW) assists in generating this proof [83].

To update the ledger, the process involves three main steps [79]:

1. Transaction Proposal and Endorsement:
  - *Proposal*: The client sends a signed transaction proposal to a peer via the gateway.
  - *Execution*: The peer executes the chaincode, signs the response, and sends it back to the gateway.
  - *Endorsement*: The gateway re-executes the transaction for endorsement, gathers signed responses, and returns a transaction envelope to the client for signing.
2. Transaction Submission and Ordering:
  - *Submission*: The client signs and submits the envelope to the gateway, which forwards it to an ordering node.
  - *Ordering*: The ordering node validates the signature, orders the transaction into a block, and sends it to all peers in the channel.
3. Transaction Validation and Commitment:
  - *Validation*: Peers validate the transaction using Idemix MSP, compare read-write sets, and check endorsement policies [80].
  - *Commitment*: Valid and invalid transactions are committed to the ledger, but only valid transactions update the world state.
  - *Commit Event*: Peers notify the client of the ledger update with a commit event [79].

In Idemix, users generate secret keys and create multiple public keys (pseudonyms) for anonymity. The Fabric Idemix implementation uses a pairing-based signature scheme for cryptographic operations [84,85]. For further details, refer to Ref. [86] and Ref. [87].

## Proposed scheme

This section provides a detailed illustration of the system architecture, the threat model, and our proposed privacy-preserving protocol.

### System model

The system consists of patients accompanied by healthcare sensors, the users’ application (UA) which provides patient information to licensed data consumers (e.g., doctors or emergency centers), gateways (GWs) such as patients’ phones or hospital access points, the healthcare application providers (APs) such as hospitals, a private Fabric BC $\left({BC}_{sensor\_auth}\right)$ containing the sensor information shared between all APs, a private Fabric BC $\left({BC}_{main}\right)$ that reduces the possibility of a mining attack, a network of peers (APs) that each peer is serving some GWs and UAs, a Fabric CA, and the Idemix MSP. Sensors gather vital patient information and transmit it anonymously to the gateways (GWs). The GWs, through the access point (AP), verify the identity of these sensors while maintaining anonymity, and then forward the encrypted data to the APs. The AP serves as the leader of an overlay network, tasked with verifying the sensors of the patients it oversees, assisting the GWs in authenticating the sensors, and providing healthcare information to registered user agents (UAs). Additionally, APs are responsible for validating transactions as peers within the Fabric blockchain.

The overlay network *i* consists of several GWs, an AP, and a private blockchain $\left(\overset{patients\_info}{\underset{i}{BC}}\right)$. Each AP maintains a blockchain for every patient referred to it, designated as ${BC}_{{Patient}_{j}}$, where *j* represents the patient's anonymous ID. Once the AP stores the encrypted data of patient *j* in its local database, it records the hash of this information in the patient's dedicated blockchain (${BC}_{{Patient}_{j}})$. Subsequently, the AP adds the hashed pointer of the last block from ${BC}_{{Patient}_{j}}$ into $\overset{patients\_info}{\underset{\;i}{BC}}$.

**Fig 4:**
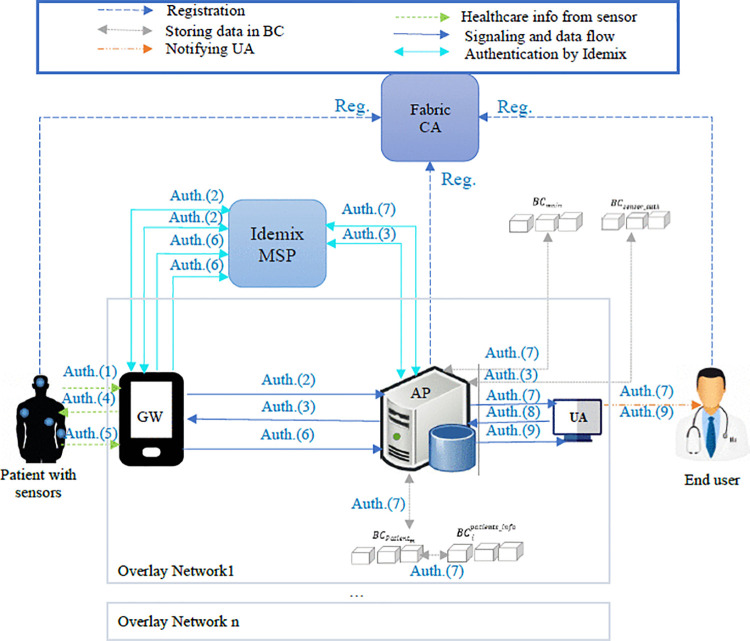
The architecture of the proposed scheme, where (*Reg.)* indicates the registration steps and *(Auth.*) denotes the authentication and data exchange steps of the protocol.

The APs and UAs register in X.509 MSP while patients and GWs register in Idemix MSP through Fabric CA (due to data and location privacy concerns related to these entities). Idemix MSP creates tokens for clients (here patients and GWs) and verifies the registered clients. The proposed system architecture is shown in Fig 4. The gateways only need to ensure the validity of the sensor without knowing the real identity of the patient. Also, the end user (for example, a doctor) does not need to know the real identity of the patient, and can make a medical diagnosis only by having the patient’s medical information. When a new health information is sent to an AP, the AP sends a notification to the end-user. Then the end-user authenticates to the AP and receives the patient’s information.

In order to adapt the Hyperledger Fabric to the context of our IoT-based healthcare system, we should attend to its scalability and high storage requirements [39,62]. Since a healthcare system contains many participants, we cluster the network to some overlay networks to improve BC scalability similar to [62]. Each overlay network uses a private BC to facilitate service provisioning to its owned nodes. The distributed nature of blockchain makes it impractical to store large volumes of data directly on it. Therefore, we store healthcare data in the database of the access points (APs) and keep only the hash of the data blocks in the private blockchain associated with the relevant overlay networks, ensuring data integrity and immutability. In our proposed scheme, to maintain patients’ anonymity and location privacy, sensors are authenticated anonymously to the gateways (GWs) via the APs, and GWs are authenticated to the APs anonymously using Idemix. Additionally, healthcare information is encrypted with the public key of the corresponding user.

To ensure distributed healthcare services and prevent single points of failure, a private blockchain (i.e., ${BC}_{sensor\_auth}$) stores sensitive patient and sensor data, such as anonymized sensor identities, anonymized sensor secret keys, hashes of one-time sensor tokens, the identities of the access points (APs) serving the anonymized patient, and the identities of authorized user agents (UAs). Access to this blockchain is restricted to authorized APs. Each AP can serve a patient's sensors regardless of prior registration. After each session, the serving AP updates the hash of the one-time token and the list of authorized UAs in ${BC}_{sensor\_auth}$. Only authorized APs can access the anonymized sensor identities and secret keys. A main private blockchain (${BC}_{main}$) is also used to mitigate 51% attacks by storing the hash of the last block from each overlay network's private blockchain (i.e.,$\;\overset{patients\_info}{\underset{\text{1}}{BC}},\overset{patients\_info}{\underset{\text{2}}{\;BC}},\ldots ,\;\overset{patients\_info}{\underset{N}{BC}}$).

Only selected and authenticated end users can access healthcare information. To facilitate this, one of the cluster heads (APs) anonymously authenticates the gateway (GW) using Idemix MSP. If the authentication is successful, the AP verifies the sensor by retrieving its anonymized identity and key based on the hash of the one-time token from ${BC}_{sensor\_auth}$. This allows the AP to authenticate both the sensor and the GW without knowing their true identities, ensuring that the actual identities of the sensors and their locations remain confidential.

The sensor authentication process in our scheme is based on a lightweight protocol inspired by Gope et al.’s approach [11]. In Gope et al.’s method, the sensor is authenticated to the GW via a local server (in this case, the AP), which prevents the GW from knowing the patient's real identity since the local server is aware of the patient's location through the serving GW. In our proposed method, the AP anonymously authenticates both GWs and sensors, and the healthcare information of patients is encrypted with the public key of the end users.

### Threat model

In our proposed method, unlike much of the existing research, all system entities, including GWs, APs, and UAs, are considered semi-honest; this means they follow the protocol correctly but may be curious about the data. Additionally, the communication channels are assumed to be insecure. Only the certificate authority (CA) is regarded as a trusted entity. An attacker can impersonate an authorized entity to read, modify, delete, or forge messages, initiate a session, and execute replay attacks. However, the attacker cannot predict a random number, obtain the private key from public-key certificates, or decrypt a message without knowing the corresponding secret key.

### Proposed protocol

This section outlines the proposed protocol, which consists of two main phases: 1) the registration phase and 2) the authentication and data exchange phase, as detailed in the following subsections.

#### Registration phase.

In this subsection, the initial registration of system entities such as patients’ sensors, UAs, APs, and GWs are presented. Two types of registration and enrollment are used in our method. One of them is the normal type where X.509 certificates are issued for UAs and APs, and the other is the anonymous type where Idemix certificates are issued for patients and GWs. Fabric CA generates its signing key pair ($\overset{CA}{\underset{pr}{k}},\;\overset{CA}{\underset{pu}{k}}$). UA and AP register in X.509 MSP while patients and GWs register in Idemix MSP through Fabric CA. Following, we present the registration phase of each entity in detail:

- **UA registration**

The end user (e.g., physician) connects to the framework through a User Application (UA). Then the UA sends the registration request to the CA. In the registration process, a registration ID and a password, are assigned to the identity of the end user. Then, the registration ID and password along with other identity-related information are stored in the CA database. Then, UA sends an enrollment request to the Fabric CA. CA issues an X.509 certificate along with the public and private key pair. The membership service provider also contains the UA’s public key, which is used to verify the validity of the signature attached to the transactions.

- **AP registration**

The AP registration process is similar to the end user registration process and APs do not need to be anonymous.

- **GW registration**

In order to preserve location privacy, the GWs should anonymously authenticate to the APs. To do this, GWs receive a certificate from the Fabric CA during registration and prove to the verifier (MSP) that they have a valid certificate using the Idemix protocol without exposing any additional information. In more detail, the GW generates its unique secret key and sends a request to the Fabric CA (issuer) to get the credentials. Credential issuance is an interactive protocol between the GW and the CA. The issuer takes its private and public keys ($\overset{CA}{\underset{pr}{k}},\;\overset{CA}{\underset{pu}{k}}$) and user attributes as inputs. Also, the user takes a user secret and the issuer’s public key, $\overset{CA}{\underset{pu}{k}},$ as inputs [80]. First, the GW sends a request, i.e., *GetCAInfo*, with an empty body to the CA to get CA’s public key, $\overset{CA}{\underset{pu}{k}}$. Also, the GW sends a NonceRequest with an empty body and an authentication header to the Fabric CA to get a nonce. The authentication header consists of an encoded username and password. Then, the CA authenticates the request, creates a random nonce, assigns an expiration time to it, stores the nonce in the database, and finally sends it to the GW. The GW generates credential requests using the CA public key ($\overset{CA}{\underset{pu}{k}}$), user secret, and the nonce sent by the CA. The request consists of a Pedersen commitment to the user’s secret (*commitment* (*userSecret*)) and a zero-knowledge proof on knowledge of the user’s secret key (*ZKProof* ($\overset{GW}{\underset{pr}{k}}$)) [80]. This commitment to the user’s secret is used as a public key. Pedersen’s commitment and zero-knowledge proof are computed according to [87]. The body of the credential request is in the form of *CredentialRequest* (*commitment* (*userSecret*)*, ZKProof* (($\overset{GW}{\underset{pr}{k}}$))*,* $\overset{CA}{\underset{pu}{k}}$*, nonce*) and the basic authentication header is in the form of basic <base64 encoding of username: password > . The GW sends the credential request to the CA.

CA validates the credential request according to the zero-knowledge proof *ZKProof* ($\overset{GW}{\underset{pr}{k}}$)*.* If the validation is successful, CA issues a GW credential by signing the commitment to the secret key in the form of ${\left[commitment\;(\overset{GW}{\underset{pr}{k}})\;\right]}_{CA}$ and stores it in the datastore [80]. Then it sends the ${credential}_{{GW}_{i}}={\left[commitment\;(\overset{GW}{\underset{pr}{k}})\;\right]}_{CA}$ and CRI back to the GW. Idemix MSP (Verifier) utilizes CRI to check whether the credentials used in a presentation token have expired or not. The GW verifies the Fabric CA's signature and stores it in his/her wallet [82]. Then GW generates a random number as ${ID}_{{GW}_{i}}$ and stores it.

- **Patient Registration**

The patient registration process is similar to the GW registration process. The patient connects to the system through the client application and sends a registration request to the Fabric CA. The CA (issuer) issues a certificate (Idemix certificate) for the patient. The patient proves to the verifier (Idemix MSP) that he/she is the owner of a valid credential using a zero-knowledge proof protocol. Since zero-knowledge proof is used, no additional information is disclosed to the verifier, issuer, or anyone else. Here, no additional information is received from the patient and the patient only interacts with the system using his/her ambiguous ID (${ID}_{Patient}$).

- **Sensor registration**

Each patient has a number of sensors that must interact with the system. For this purpose, in an offline sensor registration process, the patient generates an ID (${ID}_{s}$), a one-time token ($T_{seq}$), an array of shadow IDs ($SID=\{{sid}_{\text{1}},\;{sid}_{\text{2}},\;\ldots ,\;{sid}_{n}\}$) which is long enough, and a key for each sensor, e.g.,$\;K_{S}=({{{ID}_{s}}_{i}||n}_{h})⊕{ID}_{Patient}$ for sensor *s* where $n_{h}$ and $r_{j}$ are random numbers and ${ID}_{Patient}$ is the patient ID (obtained during patient registration). The value of ${sid}_{j}$ is $h\left({{ID}_{s}}_{i}∥r_{j}∥K_{S}\right)$ [11]. Moreover, to verify the sensor authentication, the obfuscated ID of the sensor (${AmbiguousID}_{S}$), the obfuscated key of the sensor (${AmbiguousK}_{S}$), an array of the hash values of SID ($HSID=\{h({sid}_{\text{1}}),\;h({sid}_{\text{2}}),\;\ldots ,h({sid}_{n})\}$), and the hash of one-time token of the sensor (${verfy}_{index}=hash(T_{seq})$) are stored in ${BC}_{sensor\_auth}$ for each sensor. The values of ${AmbiguousID}_{S}$ and ${AmbiguousK}_{S}$ are according to the values of (${ID}_{s}$) and ($K_{s}$) respectively and random numbers ($R_{\text{1}}$, $R_{\text{2}}$, $R_{\text{3}}$, and $R_{\text{4}}$) are calculated according to ${AmbiguousID}_{S}=\;{ID}_{s}⊕R_{\text{1}}⊕R_{\text{2}}$ and ${AmbiguousK}_{S}=\;K_{s}⊕R_{\text{3}}⊕R_{\text{4}}$. The sensors store the values of ${ID}_{s}$,$\;K_{s}$, $T_{seq}$, ${AmbiguousID}_{S}$, and ${AmbiguousK}_{S}$ in non-volatile memory.

#### Authentication phase.

**Fig 5:**
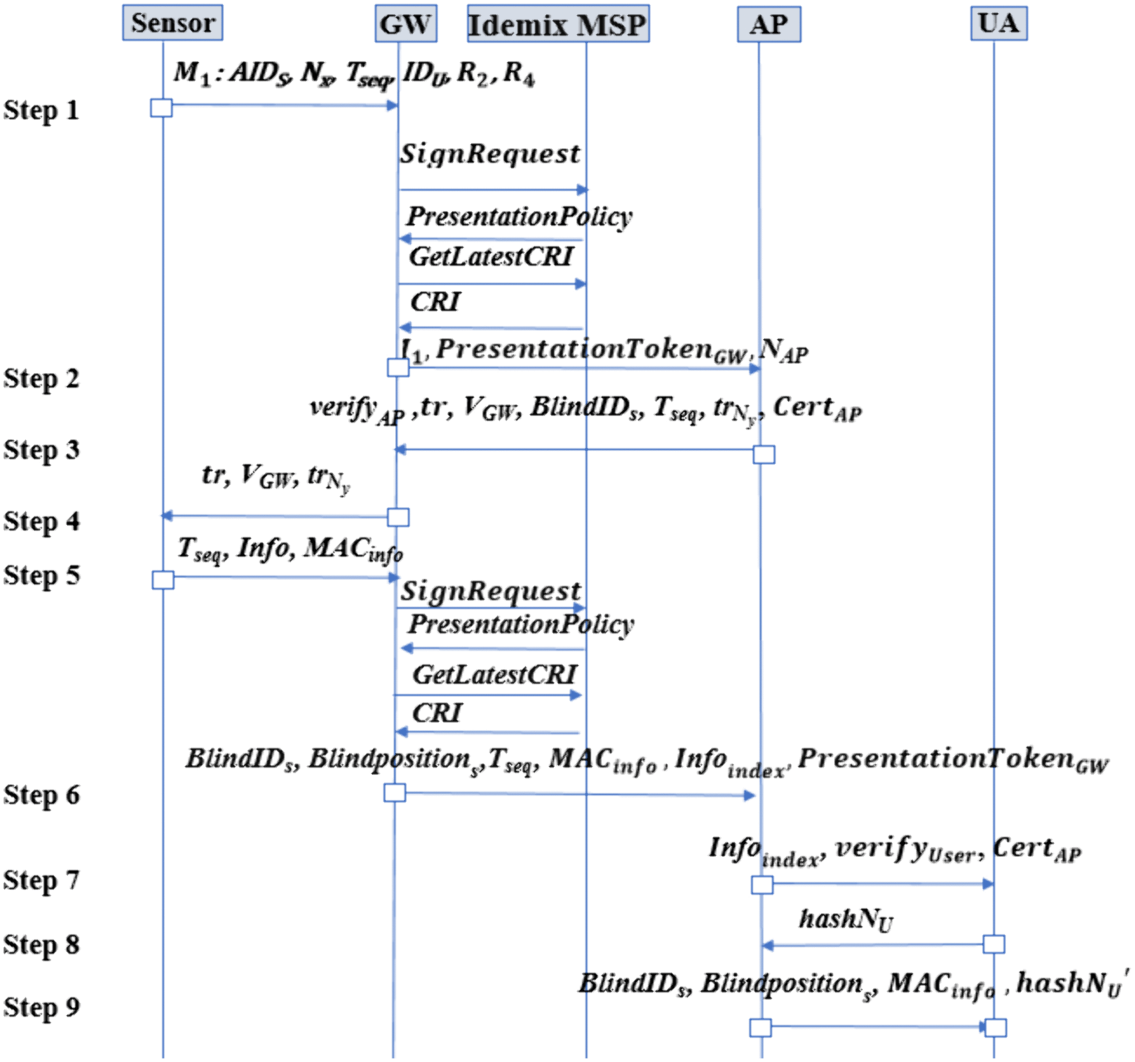
Authentication and real-time data transmission (The small rectangles represent the operations performed by the entities at each step, which have been omitted for simplicity in the protocol description. The details of these operations are provided in Figs 6–15.).

The authentication phase involves the anonymous authentication of sensors to the GWs via Idemix, the anonymous authentication of GWs to the APs using Idemix, and mutual authentication between the UAs and the APs. Our scheme is designed with consideration for the computational and energy limitations of the sensors. The AP initially authenticates the GWs through Idemix and subsequently assists the GWs in anonymously authenticating the sensors. This process ensures that the AP cannot link the sensors’ anonymized identities to their respective GWs, thus preserving the patients’ location privacy. When authentication was completed, the encrypted healthcare information is stored in the AP's local database. To safeguard this information from tampering, the AP adds the hash of the encrypted data to ${BC}_{{Patient}_{j}}$, and the hash of the latest block from ${BC}_{{Patient}_{j}}$, is recorded in $\overset{patients\_info}{\underset{i}{BC}}$. Fig 5 illustrates the authentication and data exchange phase of the proposed protocol. In this figure, operational details performed by the entities have been omitted and only transactions are shown, to facilitate understanding of the overall protocol. These details are described in below steps:

- **Step 1**

**Fig 6:**
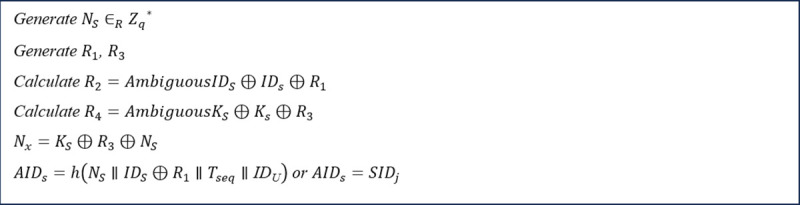
Step 1 of the proposed protocol executed by the sensors.

In the first step, the sensor generates several random numbers: $N_{S}$, $R_{\text{1}}$, and $R_{\text{3}}$. It then computes $R_{\text{2}}$ and $R_{\text{4}}$ using ${AmbiguousID}_{s}$ and ${AmbiguousK}_{s}$ with the following formulas: $R_{\text{2}}={AmbiguousID}_{s}⊕{ID}_{s}⊕R_{\text{1}}$ and $R_{\text{4}}={AmbiguousK}_{s}⊕K_{s}\;⊕R_{\text{3}}$. Additionally, the sensor calculates $N_{x}$ as $N_{x}=K_{S}⊕R_{\text{3}}{⊕N}_{S}\;$using its key, $K_{S}$. Next, it computes ${AID}_{s}$ as ${AID}_{s}=h\left(N_{S}∥{ID}_{S}⊕R_{\text{1}}∥T_{seq}∥{ID}_{U}\right)$, where ${ID}_{S}$, $\;T_{seq}$, and ${ID}_{U}$ represent the original identity of the sensor, the one-time token of the sensor, and the identity of the UA receiving the healthcare information, respectively. Fig 6 shows the operation performed by the sensor in this step.

As seen in Fig 5, after the operation performed in Fig 6, the sensor sends the message $M_{\text{1}}$ including ${AID}_{S},\;N_{x},\;T_{seq},{{\;ID}_{U},\;R_{\text{2}},\;\text{and}\;R}_{\text{4}}$ to the serving GW. Moreover, when the synchronization of the AP and the sensor is lost, the sensor should send the shadow ID (${SID}_{j}$) instead of ${AID}_{s}$. Moreover, after each use, the values of $T_{seq}$ are updated through the value of *tr* as will be discussed later in Step 5.

- **Step 2**

As seen in Fig 5, this step consists of some exchanges as below:

i Transaction Signing and Verification

Whenever a GW wants to send a transaction, it needs to create a non-revocation proof, a pseudonym, and a presentation token to prove that it is a valid GW. To do this, the GW sends a *SignRequest* to the Idemix MSP. Idemix MSP sends the presentation policies to the GW. A presentation policy specifies the information that the GW has to expose to the Idemix MSP. This information can define which credentials from which trusted Fabric CAs are required. Moreover, it defines the public keys of credential issuing authorities which the Idemix MSP trusts [88]. Also, the GW should get new revocation information from the Revocation Authority [82]. The latest CRI can be requested by sending the *GetLatestCRI* request to relevant API endpoint (Fig 5).

**Fig 7:**
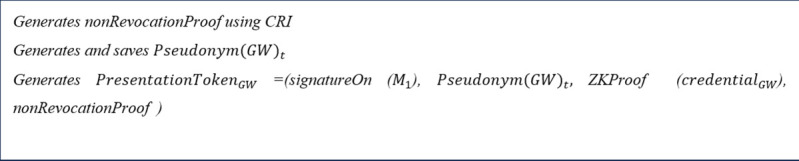
Step 2 of the proposed scheme executed by GWs.

After that, as shown in Fig 7, the GW generates a non-revocation proof that its credential has not been revoked according to the CRI [83]. After that, the GW generates a new scope-exclusive pseudonym, ${Pseudonym({GW}_{i})}_{t}$ as follows.

ii Creation of Scope-exclusive pseudonym (public key of GW)

A scope-exclusive pseudonym is unique for a secret key of the GW and a specified scope string. Moreover, scope-exclusive pseudonyms are unlinkable for different scope strings. Scope-exclusive pseudonym (public key of the GW) is the output of a verifiable random function. This function uses the scope string as input and the secret key as seed. The $T_{seq}$ can be used as the scope string [88].

iii Creation of presentation token

The GW generates a fresh presentation token from its credentials according to the presentation policy using the Idemix crypto library. The presentation token signs the content of the transaction, proof of having a valid credential issued by the CA, and defines the required attributes by the presentation policy.

By receiving the presentation policy, the GW invokes the Idemix crypto library. Then, the Idemix crypto library checks the GW for having required credentials and pseudonyms. This output specifies all the possible combinations of the GW’s credentials and pseudonyms that comply the policy. The GW selects an identity that lets the GW to select the preferred combination of credentials and pseudonyms (when there are several ways to satisfy the policy) and expresses its consent to the disclosure of its information. The GW’s selection options are stored in an object and forwarded to the Idemix crypto library. Idemix crypto library is responsible to create and return a presentation token, ${PresentationToken}_{{GW}_{i}}$ according to the GW’s selection [88]. ${PresentationToken}_{{GW}_{i}}$ contains the signature on the message, i.e., *signatureOn*(*message*), a zero-knowledge proof of the credential, i.e., *ZKProof*(${credential}_{{GW}_{i}}$), the non-revocation proof, and the GW’s pseudonym, i.e., ${Pseudonym({GW}_{i})}_{t}$ (Fig 7).

iv Mutual Authentication with the AP

Then as shown in Fig 5, to perform mutual authentication with the AP, the GW generates a random number, $N_{AP}$, and sends it along with the message $M_{\text{1}}$ received from the sensor and the ${PresentationToken}_{{GW}_{i}}$ as a signed transaction proposal to the AP.

- **Step 3**

**Fig 8:**
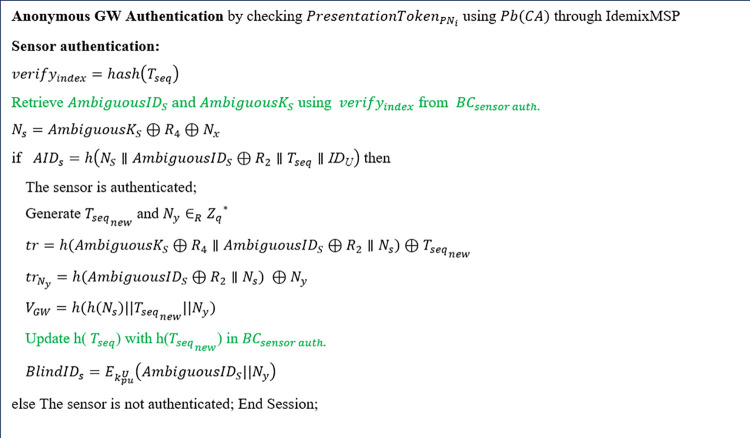
Step 3 of the proposed scheme executed by AP (Green texts indicate operations on the blockchain).

Step 3 involves the anonymous authentication of the GW to the AP, the authentication of the sensor to the AP, updating the sensor’s token, encrypting the sensor's identity for the UA, and the authentication of the AP to the GW, as detailed below (Fig 8).

i Anonymous authentication of the GW to the AP (Verification of the presentation token)

When the AP receives the presentation token from the GW, the AP anonymously authenticates the GW through the Idemix MSP. The Idemix MSP forwards it to the Idemix crypto library to check whether the presentation token, ${PresentationToken}_{{GW}_{i}}$, matches the corresponding presentation policy alternatives. The token validation is done in two phases. At the first, it checks if the statements of the presentation token (${PresentationToken}_{{GW}_{i}}$) provide the required statements of presentation policy. Second, it verifies the given token description [88]. ${PresentationToken}_{{GW}_{i}}$ is verified with the help of the CA’s public key that signed the credential [80]. Idemix MSP verifies the signature, the non-revocation proof, and zero-knowledge proof of the credentials, i.e., *ZKProof* (${credential}_{{GW}_{i}}$)*.*

ii Authentication of the sensor to the AP

The AP authenticates the sensor using the information stored in ${BC}_{sensor\_auth}$. To do this, the AP retrieves ${AmbiguousID}_{s}\text{ and }{AmbiguousK}_{s}$ from ${BC}_{sensor\_auth}$ by hashing the sensor's one-time token, specifically ${verify}_{index}=hash\left(T_{seq}\right)$. The AP then computes the value of $N_{s}$ as $N_{s}={AmbiguousK}_{s}⊕R_{\text{4}}⊕N_{X}$, using the nonce $N_{x}$ received from the GW. The AP verifies the sensor by comparing the ${AID}_{s}$ received from the GW with the ${AID}_{s}$ it calculates as $h\left(N_{S}∥{AmbiguousID}_{s}⊕R_{\text{2}}∥T_{seq}∥{ID}_{U}\right)$. If these values do not match, the AP terminates the session and notifies the GW that the sensor authentication has failed. If they match, the sensor is authenticated, and the AP proceeds to update the sensor's token.

iii Updating the sensor’s token

Once the AP authenticates the sensor, it generates a new one-time token, ${T_{seq}}_{new}$, and computes $tr=h({AmbiguousK}_{s}⊕R_{\text{4}}∥{AmbiguousID}_{s}⊕R_{\text{2}}∥N_{s})⊕{T_{seq}}_{new}$. This value is sent to the sensor via the GW, along with $V_{GW}=h(h(N_{s})∥{T_{seq}}_{new}||N_{y})$, which the sensor uses to verify the integrity of the new token. Additionally, the AP calculates ${tr}_{N_{y}}=h({AmbiguousID}_{s}⊕R_{\text{2}}∥N_{s})⊕N_{y}$ and forwards it to the sensor through the GW, where $N_{y}$ is a random number used by the sensor to compute ${MAC}_{info}$ in step 5. The AP also updates ${verify}_{index}$ to ${verify}_{index}=hash{{(T}_{seq}}_{new})$ in ${BC}_{sensor\_auth}$, allowing other APs to access this value.

iv Encrypting the Sensor's Ambiguous Identity for the UA

In this step, the AP generates ${BlindID}_{s}=E_{\overset{U}{\underset{pu}{K}}}\left({AmbiguousID}_{s}∥N_{y}\right)$, which will be sent to the UA in step 9. This ensures that the ambiguous identity of the sensor (${AmbiguousID}_{s})$ and $N_{y}$, used to verify the integrity of the patient’s information, are only exposed to the UA.

v Authentication of the AP to the GW

In this step, the AP encrypts a nonce, ${\text{N}}_{AP}$, received from the GW using its private key, resulting in ${verify}_{AP}=E_{\overset{AP}{\underset{pr}{k}}}({\text{N}}_{AP}∥T_{seq})$.

After the operation illustrated above (Fig 8), as shown in Fig 5, the AP sends ${verify}_{AP}$, $tr,\;V_{GW},\;{BlindID}_{s}$*,* $T_{seq}$, ${tr}_{N_{y},}\;$ and ${Cert}_{AP}$ to the GW, enabling the GW to verify the AP through its signature on the nonce

- **Step 4**

**Fig 9:**
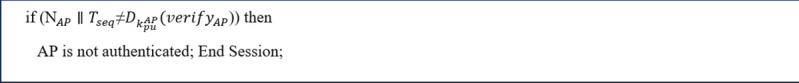
Step 4 of the proposed scheme executed by GWs.

In this step, the AP authenticates itself to the GW using the ${verify}_{AP}$ value received from the AP. As shown in Fig 9, the GW checks if decrypting ${verify}_{AP}$ yields $N_{AP}∥T_{seq}$, where $N_{AP}$ was generated in step 2 and $T_{seq}$ was received from the sensor in step 1. If the values do not match, the session is terminated otherwise the GW authenticates the AP and temporarily stores ${BlindID}_{s}$ for later transmission to the AP. Then as shown in Fig 5, the GW sends $V_{GW},\;tr$, and ${tr}_{N_{y}}$ to the sensor.

- **Step 5**

**Fig 10:**
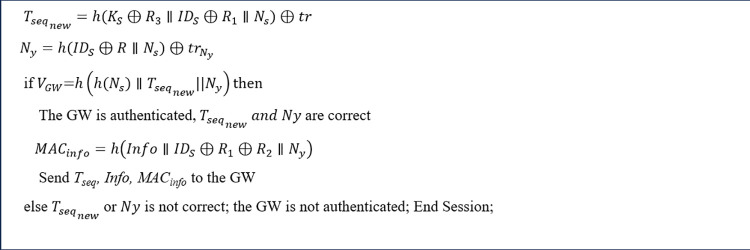
Step 5 of the proposed scheme executed by sensors.

As shown in Fig 10, the sensor computes the new token value as ${T_{seq}}_{new}=h(K_{S}⊕R_{\text{3}}∥{ID}_{S}⊕R_{\text{1}}∥N_{s})⊕tr$. Next, the sensor calculates *Ny* using ${tr}_{N_{y}}$ with the formula $N_{y}=h({ID}_{S}⊕R_{\text{1}}∥N_{s})⊕{tr}_{N_{y}}$. The sensor then authenticates the GW by verifying $V_{GW}=h(h(N_{s})∥{T_{seq}}_{new}||N_{y}$), which confirms the integrity of the new token and $N_{y}$. After that, the sensor generates the authentication code ${MAC}_{info}=h(Info∥{ID}_{s}⊕R_{\text{1}}⊕R_{\text{2}}∥N_{y})$ to ensure the integrity of the healthcare information (*Info*) that will be sent to the UA.

Then, as shown in Fig 5, the sensor transmits $T_{seq},\;{MAC}_{info}$, and *Info* to the GW, updating the one-time token to ${T_{seq}}_{new}$.

- **Step 6**

As shown in Fig 5, the GW performs the initial two phases of step 2 to authenticate itself anonymously to the AP.

**Fig 11:**
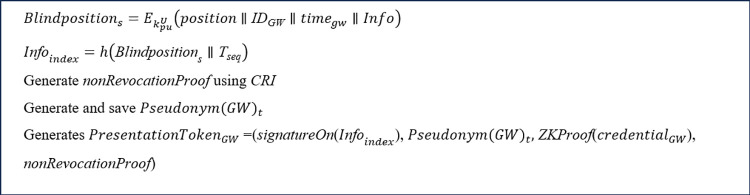
Step 6 of the proposed scheme executed by GWs.

Then, as shown in Fig 11, the GW encrypts its location (*position*), its identity (${ID}_{GW}$), and the healthcare information (*Info*) received from the sensor in step 5 using the UA's public key, resulting in ${Blindposition}_{s}=E_{\overset{U}{\underset{pu}{k}}}(position∥{\text{ID}}_{\text{GW}}∥{time}_{gw}∥Info)$. This ${Blindposition}_{s}$ allows the UA to locate the patient in emergencies. The GW then computes ${Info}_{\text{index}}=h({Blindposition}_{s}∥\;T_{seq})$. Next, the ${PresentationToken}_{\text{GW}}=(signatureOn(message)$*,* ${Pseudonym(GW)}_{t}$, *ZKProof* (${credential}_{GW}$)*, non-revocation proof*) is generated in a manner similar to the third phase of step 2.

Finally, as shown in Fig 5, the GW sends ${BlindID}_{s}$, ${Blindposition}_{\text{s}}$, $T_{seq}$, ${MAC}_{info}$, ${Info}_{\text{index}}$, and ${PresentationToken}_{\text{GW}}$ to the AP.

- **Step 7**

**Fig 12:**
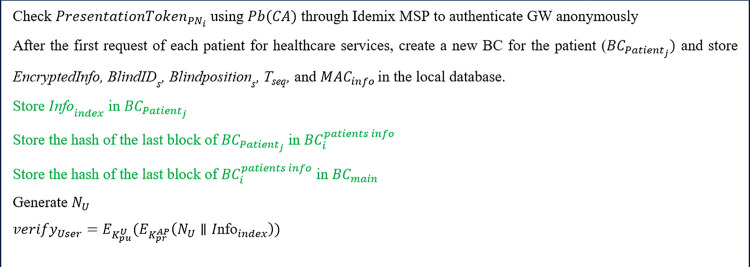
Step 7 of the proposed scheme executed by Aps.

In this step, as shown in Fig 12, the AP first anonymously authenticates the GW using Idemix MSP, similar to the first phase of step 3, by verifying the ${PresentationToken}_{\text{GW}}$ with the CA's public key. The AP then adds the patient’s information, specifically ${Info}_{index}$, to the blockchain for ${Patient}_{j}$. This blockchain is established for ${Patient}_{j}$ after the AP receives the initial request from them. Additionally, the AP stores the patient’s details, including ${EncryptedInfo,\;BlindID}_{s},$ ${Blindposition}_{s},\;{T_{seq},\;\text{and}\;MAC}_{info\;}$, in its local database. Following this, the index of the blockchain for ${Patient}_{j}$ and the hash of its most recent block are recorded in $\overset{patients\_info}{\underset{i}{BC}}$, while the hash of the last block from $\overset{patients\_info}{\underset{i}{BC}}$ is saved in ${BC}_{\text{main}}$.

To facilitate end-user authentication, the AP generates a random number, $N_{U}$, signs the combination of ${Info}_{index}$ and $N_{U}$ using its private key, and encrypts the result with the UA’s public key, resulting in ${{verify}_{User}=E_{\overset{U}{\underset{pu}{K}}}(E}_{\overset{AP}{\underset{pr}{K}}}(N_{U}∥{Info}_{index})).$

Finally, as shown in Fig 5, the AP informs the UA by sending ${verify}_{User}$, ${\;Info}_{index}$and ${Cert}_{AP}.$ The values of ${BlindID}_{s}$ and ${BlindPosition}_{s}$ are encrypted using the UA’s public key to safeguard the privacy of healthcare information and the patient’s location, which could be compromised if the serving GW’s identity were disclosed.

- **Step 8**

**Fig 13:**
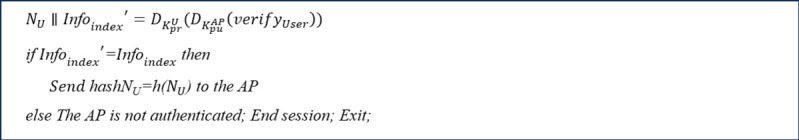
Step 8 of the proposed scheme executed by UAs.

In this stage, as shown in Fig 13, the UA authenticates the AP by using the AP's certificate to decrypt the value of ${verify}_{User}$, which allows the UA to retrieve both the ${Info}_{index}$ and $N_{U}$.

The UA then calculates the hash of $N_{U}$, referred to as ${hashN}_{U}$, and transmits it to the AP. Since only the UA can derive $N_{U}$ from ${verify}_{User}$, it sends ${hashN}_{U}$ to the AP to complete the authentication process (Fig 5).

- **Step 9**

**Fig 14:**
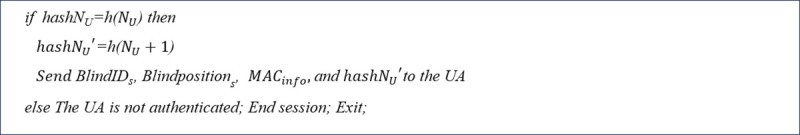
Step 9 of the proposed scheme executed by Aps.

In this step, as shown in Fig 14, the AP verifies the UA by comparing ${hashN}_{U}$ to $h\left(N_{U}\right)$. If the authentication succeeds, the AP computes ${{hashN}_{U}}^{\prime }=h(N_{U}+\text{1})$ and as shown in Fig 5, sends the values of ${BlindID}_{s}$, ${Blindposition}_{s},$ ${MAC}_{info}$, and ${{hashN}_{U}}^{\prime }$ to the UA.

**Fig 15:**
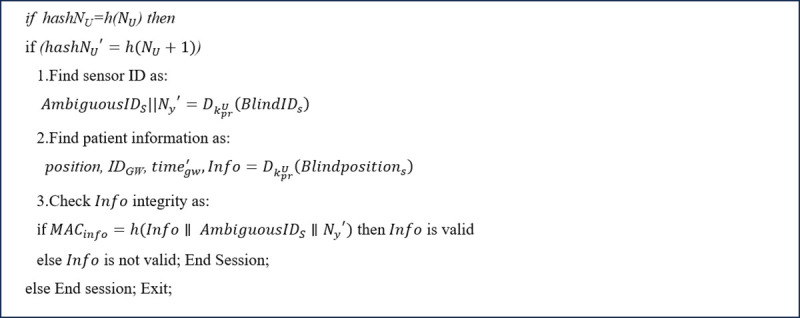
Step 10 of the proposed scheme executed by UAs.

Then, as shown in Fig 15, initially, the UA authenticates the AP using the value of ${{hashN}_{U}}^{\prime }$. It then retrieves sensor information through the following steps:

1. The UA decrypts ${BlindID}_{s}$ to obtain the ambiguous identity of the sensor (${AmbiguousID}_{S}$) and ${N_{y}}^{\prime }$ (i.e.,$\;{AmbiguousID}_{S}||{N_{y}}^{\prime }=D_{\overset{U}{\underset{pr}{k}}}\left({BlindID}_{s}\right)$) which is used to verify the integrity of healthcare information.
2. The UA decrypts ${Blindposition}_{s}$ to obtain the gateway's location (*position*)*,* the gateway's identity (${ID}_{GW}$), the timestamp $\left(\overset{\prime }{\underset{gw}{time}}\right)$*,* and healthcare information (*Info*) (i.e., $position∥{ID}_{GW}∥\overset{\prime }{\underset{gw}{time}}∥Info=D_{\overset{U}{\underset{pr}{K}}}\left({Blindposition}_{s}\right))$).
3. The UA ensures the integrity of the information by comparing the received ${MAC}_{info}$ to $h(Info∥{AmbiguousID}_{S}∥{N_{y}}^{\prime })$. Thus, the UA is the only entity with access to the healthcare information and location, while only the ambiguous identities of the sensors are accessible to it.

1) Patient data retrieval by the ENd users

**Fig 16:**
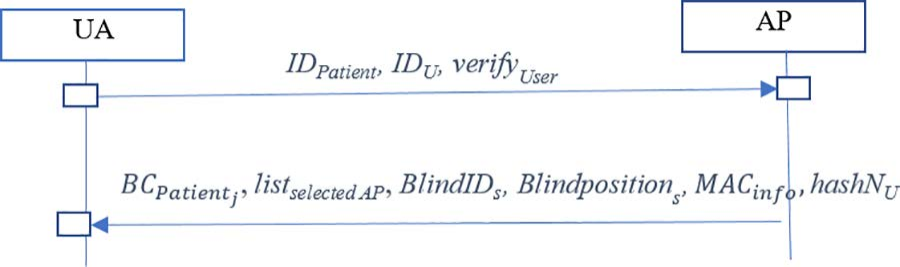
Patient data retrieval by the UA.

**Fig 17:**
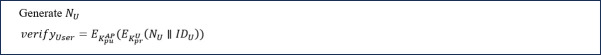
Step 1 of data retrieval phase executed by UAs.

Fig 16 shows the proposed protocol for UAs to access patients’ healthcare data in three steps:

Step1: A UA initiates access by sending a request to any AP that includes the UA’s identifier (${ID}_{U}$), the patient’s identifier (${\text{ID}}_{\text{Patient}}$) and ${verify}_{User}$ (as described in step 7 of the authentication phase) (see Fig 17).

**Fig 18:**
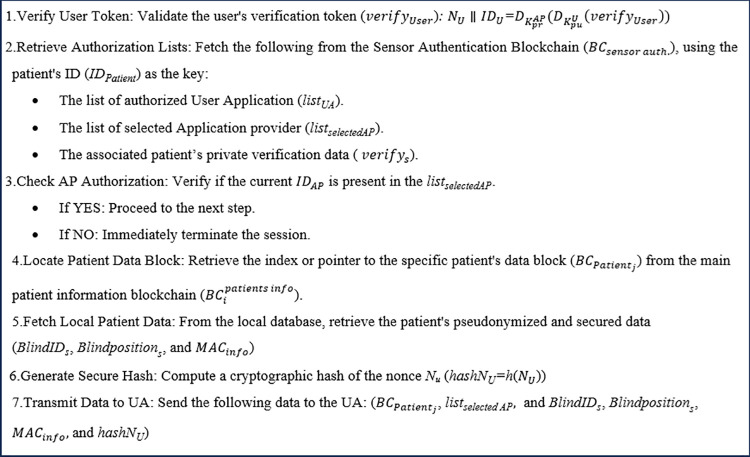
Step 2 of data retrieval phase executed by APs.

Step 2: Upon receiving the request, the AP authenticates the UA by decrypting ${verify}_{User}$ with the AP’s private key. If authentication succeeds, the AP obtains from ${BC}_{sensor\_auth}$ the selected-AP list for the patient (${\text{list}}_{selected\;AP}$), the list of authorized UAs (${list}_{UA}$), and the patient’s private verification data (${\;verify}_{s}$) to determine whether the UA is permitted to access the patient’s records via this AP. If the AP appears in ${list}_{selected\;AP}$, it uses the index from $\overset{patients\_info}{\underset{i}{BC}}$ to retrieve the patient’s stored data (e.g., ${BlindID}_{s}$, ${Blindposition}_{s}$*,* and ${MAC}_{info}$) from its local database. The AP computes ${hashN}_{U}=h(N_{U}$*)* and returns this value together with the selected-AP list to the UA (Fig 18).

Step3: On receipt, the UA verifies the AP by checking ${hashN}_{U}$ and then decrypts the patient data as described in Step 10 of previous section. The UA may repeat this process to request the patient’s information from other APs in the selected list. Patients may also access their own medical records using the UA role (like Fig 15).

## Evaluation

The subsequent subsections discuss the security and performance attributes of the proposed method and compare it to traditional approaches.

### Performance evaluation

This section presents a comparison of the proposed method's performance against the approaches outlined in Refs. [25,51,89]. Subsequently, the analysis focuses on computation and communication overhead, latency, and the energy consumption of sensors. These metrics are assessed based on the configurations specified in Table 1 for each system component. Computation overhead is determined by measuring the execution times of encryption processes on system entities such as GWs, APs, and sensors, as detailed in Table 2. Furthermore, the execution times for blockchain operations—reported in Table 3—are obtained from Hyperledger Fabric version 1.4.0, under the system setup described in [90]. The issuance and verification times are recorded as 1766 ms and 733 ms, respectively, utilizing the Idemix protocol set and assuming a single attribute, as noted in [91].

**Table 1:** System configurations considered for evaluating the proposed method.

Entity	System description
GWs and UAs	NanoPi Fire3 which uses the Samsung 64-bit Octa-core Cortex-A53 S5P6818 SoC with 1-GB DDR3 RAM [92]
APs and KDC	An Intel Core i7-4702MQ processor and 16-GB RAM [92]
WBAN sensors	Mica2 [93–96]
BC	Hyperledger Fabric 1.4.0 [90]

**Table 2:** Execution time of security primitives on sensors, GWs, and servers.

Symbol	Description	Execution time on the sensor (Mica2 [93–96])	Execution time on GW and UA (nanoPi fire [92])	Execution time on AP, Idemix MSP, and KDC (Intel core-i7 [92])
$T_{RN}$	The execution time of random generation	0.44ms	Negligible	Negligible
$T_{XOR}$	The execution time of XOR operation	0.001ms	Negligible	Negligible
$T_{hash}$	The execution time of SHA1 (16 bytes)	3.63ms	1.7 μs	235ns
$T_{mul}$	The execution time of the ECC point multiplication operation (SECP 160R1)	0.81s	475 μs	46 μs
$T_{add}$	The execution time of point addition operation (SECP 160R1)	3.375ms	2 μs	192 ns
$T_{ENC-ECC}$	The execution time of public-key encryption 160bit ECDSA (SECP 160R1)	0.813s	0.6 ms	61 μs
$T_{DEC-ECC}$	The execution time of public-key decryption 160bit ECDSA (SECP 160R1)	1.623s	2.1 ms	228 μs
$T_{AES}$	AES-128 CBC (16 Bytes)	–	409 ns	141 ns

**Table 3:** Execution time of blockchain operations on hyperledger (smart contract asset size = 100 bytes) [90].

Symbol	BC name	State DB	Smart Contract Method	Execution time (s)
$T_{StoreToSensor\_authBC}$	${BC}_{sensor\_auth}$	couchDB	createAsset	0.22
$T_{RetrieveFromSensor\_authBC}$	${BC}_{sensor\_auth}$	couchDB	getAsset	0.06
$T_{StoreToPatientBC}$	${BC}_{{Patient}_{j}}$	levelDB	createAsset	0.11
$T_{RetrieveFromPatientBC}$	${BC}_{{Patient}_{j}}$	levelDB	getAsset	0.05
$T_{StoreToPatients\_infoBC}$	$\overset{patients\_info}{\underset{\;i}{BC}}$	levelDB	createAsset	0.11
$T_{RetrieveFromPatients\_infoBC}$	$\overset{patients\_info}{\underset{\;i}{BC}}$	levelDB	getAsset	0.05
$T_{StoreToMainBC}$	${BC}_{main}$	levelDB	createAsset	0.11
$T_{RetrieveFromMainBC}$	${BC}_{main}$	levelDB	getAsset	0.05
$T_{StoreToBC(\text{couchDB})}$	BC	couchDB	createAsset	0.22
$T_{RetrieveFromBC(\text{couchDB})}$	BC	couchDB	getAsset	0.06
$T_{RetrieveFromBC-batch(\text{couchDB})}$	BC	couchDB	batchGetAsset	0.18

#### Computation overhead evaluation.

The computational overhead for each system entity is detailed in Table 4 and compared to the overhead of the methods described in refs. [25,51,89]. Reference [25,51] are our previous work. Ref. [25] offers a centralized, end-to-end privacy-preserving solution. In contrast, Ref. [51] presents a blockchain-based end-to-end privacy method. In addition, Ref. [89] presents a blockchain-based distributed privacy-preserving method for healthcare systems, which includes (1) sensors and the patient's smartphone, (2) an IoT health manager (IHM) that receives data from the smartphone and stores the hash of that information in the blockchain, (3) the blockchain and miners, (4) healthcare servers that initiate the primary blockchain, manage clusters, and register other entities, and (5) a healthcare wallet. Ref. [89] uses two separate chains—one for access policies and another for data—and assumes all entities are trusted while considering a secure channel between the sensors and the patient's smartphone. In this setup, the smartphone gathers information from the sensors, encrypts it with a symmetric key, and sends the encrypted data to the IHM. The IHM then analyzes the data and sends its hash to one of the miners, who checks the transaction cluster and forwards it to all miners in that cluster. Each miner verifies the transaction and adds it to the blockchain. Additionally, the IHM calculates and stores the access policy transaction in the policy chain for healthcare staff access control.

**Table 4:** Comparing the computation overhead of the proposed scheme with the approaches in refs. [25,51,89] (n: the number of shadow ids = 100, n: the number of aps = 10, and s: seg-size = 10).

Scheme	Entities	Computation overhead	Time (ms)
Proposed scheme	Sensor	$\text{9}\times T_{XOR}+\text{7}\times T_{RN}+\text{6}\times T_{hash}$	≈24.869ms
	GW	$\text{2}\times T_{Issuance}+\text{1}\times T_{ENC}+\text{1}\times T_{DEC}+\text{1}\times T_{hash}$	≈3534.70 ms
	AP	$\text{2}\times T_{verification}+\text{8}\times T_{hash}+\text{1}{\times T}_{RetrieveFromSensor\_authBC}+\text{8}\times T_{XOR}+\text{3}\times T_{RN}+\text{1}\times \;T_{StoreToSensor\_authBC}+\text{4}\times T_{ENC}\;\;\;\;+\text{1}\times T_{StoreToPatientBC}+\text{1}\times T_{StoreToPatients\_infoBC}+\text{1}\times T_{StoreToMainBC}$	≈2076.24 ms
	UA	$\text{3}\times T_{DEC}+\text{3}\times T_{hash}$	≈6.3051 ms
Method of [25](2021)	Sensor	$\text{1}\times T_{RN}+\text{3}\times T_{XOR}+\text{6}\times T_{hash}$	≈22.223
	GW	$\text{4}\times T_{RN}+\text{6}\times T_{mul}+\text{2}\times T_{hash}+\text{2}\times T_{add}+\text{2}\times T_{ENC}+\text{1}\times T_{DEC}$	≈6.1574
	NM	$\text{2}\times T_{RN}+\text{3}\times T_{mul}+\text{1}\text{0}\times T_{hash}+\text{4}\times T_{pairing}+\text{3}\times T_{XOR}+\text{5}\times T_{ENC}$	≈2.229
	KDC	$\text{1}\times T_{ENC}$	≈0.061
	AP	$\text{5}\times T_{DEC}+\text{1}\times T_{add}+\text{1}\times T_{hash}$	≈1.14
	User	$\text{1}\times T_{RN}+\text{2}\times T_{ENC}+\text{3}\times T_{DEC}+\text{2}\times T_{hash}$	≈7.5
Method of [89] (2021)	Patient cellphone	$T_{AES}$	≈0.000409
	IHM	$T_{AES}+\text{2}{\times T}_{hash}+\;T_{StoreToBC(\text{couchDB})}+\text{2}\times T_{ENC-ECC}+{\text{2}\times T}_{DEC-ECC}+T_{StoreToBC(\text{couchDB})}$	≈445.404
	Miner/BC	$T_{ENC-ECC}+T_{DEC-ECC}$	≈0.289
	Healthcare staff	$T_{ENC-ECC}+T_{DEC-ECC}$	≈2.7
	IHM	$T_{hash}+\text{2}\times T_{ENC-ECC}+{\text{2}\times T}_{DEC-ECC}$	≈5.402
Method of [51] (2024)	Sensor	$T_{RN}+\text{5}\times T_{XOR}+\text{7}\times T_{hash}$	25.855
	GW	$s\times T_{GraphGeneration}+s\times T_{LSB}+s\times T_{response}+s\times T_{AES}+T_{DEC-ECC}+T_{ENC-ECC}\;+\text{1}\times T_{XOR}$	2.69 + 2.59 × s = 28.59
	AP	$T_{split}+s\;\times T_{getGi}+s\;\times T_{getRes}+s\;\times T_{LSB}+\text{1}\text{0}T_{hash}+s\times T_{response}+\text{4}\times T_{XOR}+s\times T_{AES}\;\;\;+\text{2}\times T_{DEC-GK}+\;\text{3}\times T_{RN}+T_{ENC-GK}+\text{5}T_{ENC-ECC}+T_{RetrieveFromSensor\_authBC}\;\;\;+T_{RetrieveFromUA\_authBC}+T_{StoreToMainBC}\;T_{StoreToSensor\_authBC}+T_{StoreToPatientBC}\;\;\;\;+T_{StoreToPatients\_infoBC}+T_{XOR}+(\text{2}+n)\times T_{RN}+(n+\text{1})\times T_{hash}{+T}_{ENC-GK}\;\;\;+\;T_{StoreToSensor\_authBC}+T_{solve}\;+\;T_{GraphGeneration}$	662.88 + 0.22 × s + 220.55 + 0.000235 × n = 885.6535
	UA	$\text{5}\times T_{DEC-ECC}+\text{3}\times T_{hash}$	10.50
	KDC	$T_{StoreToUA\_authBC}+T_{RN}+\;T_{MKeyGen}$	110 + 0.845 × N = 194.5

**Fig 19:**
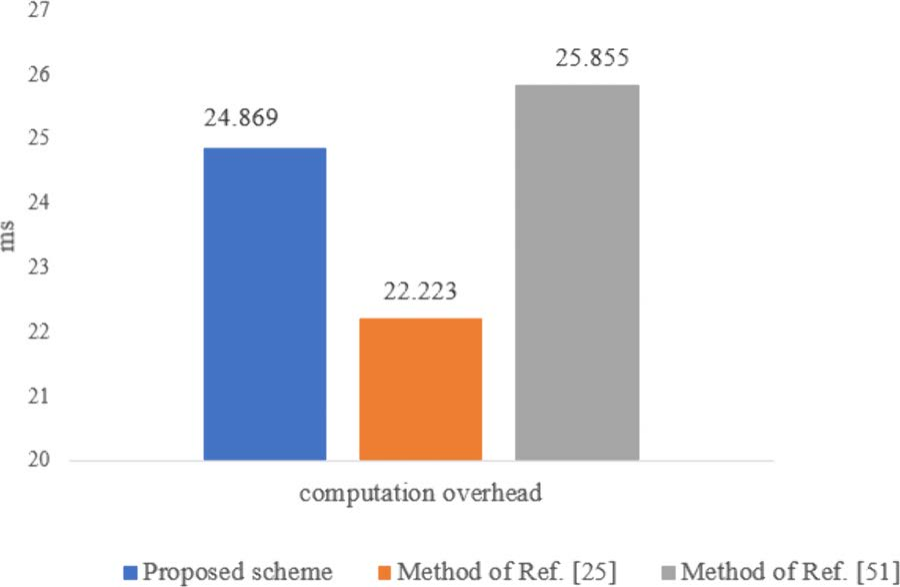
The computation overhead of sensors in the proposed scheme compared to the methods of Refs. [25,51] (for the setting with the number of shadow IDs = 100, the number of Aps = 10, and Seg-size = 10).

In our scheme, considering the computational and power limitations of sensors, we use only lightweight operations such as hashing and XOR in sensors, shifting the computational burden to the gateways. While the computation overhead imposed to sensors is near to or even lower than that of some previous studies (see Fig 19), the computation time for GWs and APs is increased due to the overhead associated with blockchain operations. However, this trade-off results in enhanced privacy and security for the blockchain-based systems, effectively addressing the single point of failure issue. During the authentication and data transmission phases, the computational overhead of our proposed method is slightly higher than that of Ref. [89].

#### Communication overhead evaluation.

**Fig 20:**
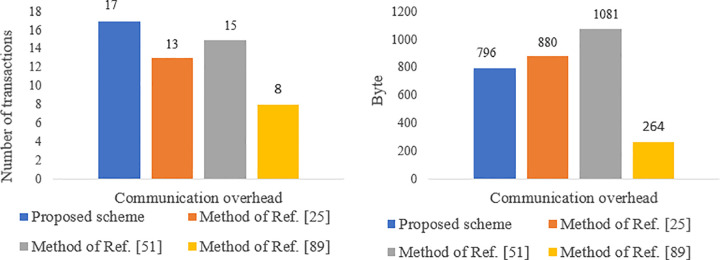
Communication overhead of the proposed scheme compared to the methods of Refs. [25,51,89].

**Fig 21:**
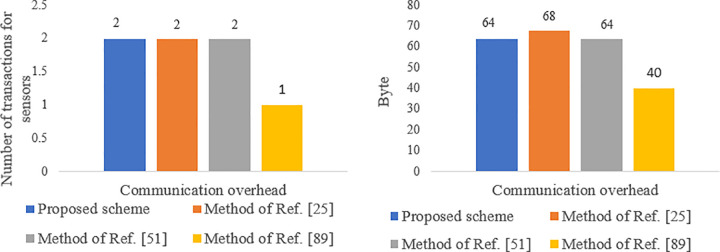
Communication overhead of sensor in the proposed scheme compared to the methods of Refs. [25,51,89].

Table 5 presents the communication overheads of the protocol, which are calculated based on the number of transactions and the volume of messages exchanged by each entity. To determine the communication overhead, the sizes of an identity, a nonce, and a one-time token are assumed to be 4 bytes each, while the sizes of healthcare information and hash values are assumed to be 20 bytes and 16 bytes, respectively. Additionally, the size of the credential is considered to be 86 bytes like Ref. [97]. Our method employs 160-bit ECDSA, AES-128 CBC (16 bytes), and SHA1 (16 bytes) for ECC encryption and decryption, AES encryption, and hashing, respectively. Given the limited bandwidth of communication channels and the energy constraints of sensors, the communication overhead for sensors is more critical than for other entities. As shown in Fig 20, the total transaction volume in our proposed method is lower than that of Refs. [51,25,89]. Furthermore, as shown in Fig 21, the communication overhead for the sensor is less than that of Ref. [25,51] but slightly higher than that of Ref. [89]. The approach in Ref. [89] only ensures privacy for part of the end-to-end communication path and assumes that all system entities are trusted, failing to adequately address issues like anonymity against internal entities, untraceability, and resilience against attacks such as collusion, mining, replay, and impersonation. Therefore, the higher communication overhead of our proposed method is justified compared to Ref. [89], given its enhanced security capabilities for patients (see Table 8).

**Table 5:** Comparison of communication overhead in the proposed scheme with the methods in refs. [25,89].

Scheme	Entity	Number of transactions	The volume of data transmitted (in bytes)
Proposed scheme	Sensor	2	64
	GW	6	236
	AP	4	360
	UA	1	16
	Idemix	4	120
	Total	17	796
[25] (2021)	Sensor	2	68
	GW	4	400
	NM	2	202
	KDC	1	86
	AP	3	100
	UA	1	24
	Total	13	880
[89] (2021)	Sensor	1	40
	Patient cellphone	1	20
	Healthcare center	–	–
	IHM	3	172
	Miner/BC	3	32
	Healthcare staff	–	–
	Total	8	264
Method of [51] (2024)	Sensor	2	64
	GW	3	297
	AP	5	414
	UA	3	48
	KDC	2	258
	Total	15	1081

**Table 8:** Comparing the proposed method to top-related work in terms of security features and resistance to attacks.

Category	Research	Privacy		Blockchain Type		Security Features											Resistance to Attack								
		Healthcare Information	Patient Location		Assumptions	Mutual Authentication	Access Control	Data Integrity	Freshness	Data Confidentiality	Anonymity against external attackers	Anonymity against internal entities	Untraceability	Availability	Privacy	Scalability	Storage	Mining	Impersonation	Replay	Man-In-The-Middle	Modification	Eavesdropping	Collusion	Single point of failure problem
Centralized	[11] (2015)	*	*	–	Some trusted entities						*				*	*			*	*					*
	[10] (2016)	*	*	–	Trusted entities	*					*		*		*				*	*	*				*
	[2] (2016)	*	*	–	Trusted entities			*	*		*				*				*						*
	[1,6] (2020)	*		–	Trusted entities	*					*		*		*				*	*	*				*
	[17] (2019)	*		–	Trusted entities	*		*	*						*	*									*
	[18] (2019)	*	*	–	Trusted entities	*		*		*					*										*
	[19] (2029)			–	Some trusted entities	*						*		*					*	*					
	[20] (2019)	*	*	–	Secure channel, Semi-trusted entities						*				*				*			*	*		*
	[21] (2020)	*		–	Secure channel, Untrusted entities		*		*	*					*							*	*		*
	[24] (2016)	*	*	–	Some trusted entities								*		*				*	*			*		
	[28] (2016)	*	*	–	Trusted entities					*	*		*		*				*	*					*
	[25] (2021)	*	*	–	Untrusted entities	*	*	*	*		*	*	*		*				*	*	*	*			*
BC-based	[60] (2020)	*		Not specified	–		*								*	*	*					*			
	[59] (2020)	*		Public	Trusted entities			*		*				*	*		*								
	[29] (2016)	*		Consortium	Trusted entities	*	*			*					*	*						*			
	[30] (2017)	*		Private	Untrusted entities	*	*	*		*				*	*							*			
	[31] (2017)	*		Not defined	–	*	*	*						*	*	*									
	[39] (2018)	*		Private	Trusted entities		*			*	*				*	*	*							*	
	[41] (2019)	*		Private	Some trusted entities		*	*				*			*							*			
	[62] (2019)	*		Not defined	Trusted entities	*	*	*		*	*	*		*	*	*	*	*							
	[61] (2020)	*		Private	Trusted entities	*	*			*					*		*					*			
	[55] (2020)	*		Private,Public	–		*								*	*						*	*		*
	[56] (2020)	*		Private	Trusted entities		*			*				*	*	*						*			
	[5,8] (2019)	*		Public	Secure channel, Trusted entities		*			*					*	*	*					*	*		
	[89] (2021)	*		Consortium	Trusted entities	*	*	*		*	*				*							*	*		
	[51] (2024)	*	*	Private,Public	Untrusted entities	*	*	*	*		*	*	*	*	*	*	*	*	*	*	*		*		
	[101] (2022)	*		Public	Untrusted entities	*	*			*	*	*		*	*				*		*	*			
	[102] (2023)	*		Public	Untrusted entities	*	*	*		*				*	*			*	*			*	*		
	Our scheme	*	*	Private	Untrusted entities	*	*	*	*	*	*	*	*	*	*	*	*	*	*	*	*	*		*	

#### Latency evaluation.

**Fig 22:**
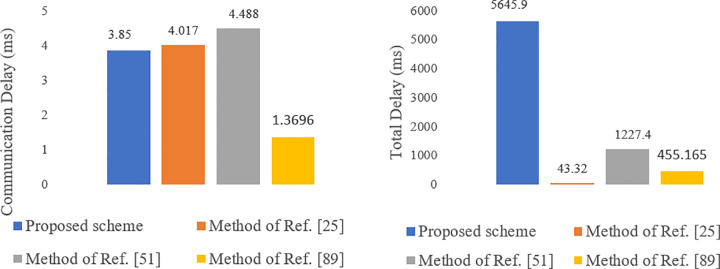
Communication delay and total end-to-end delay of the proposed scheme compared to the methods of Refs. [25,51,89].

Latency refers to the time it takes to transfer a healthcare data packet from a sensor to a UA [1]. This end-to-end latency encompasses both computing delay and communication delay. The computing delay is the total time taken for operations performed by each system entity according to the proposed protocol (as shown in Table 4). For communication delay, we assume a 250 kbps wireless link for connections between sensors and gateways (GWs), and a 20 Mbps broadband WAN link for connections among other entities. Additionally, queuing and routing delays are considered negligible. The communication delay is analyzed during the authentication and data transmission phases in comparison to Refs. [25,51,89]. Table 6 presents a comparison of the computation delay, communication delay, and total end-to-end delay of our proposed scheme with those of Refs. [25,51,89]. As shown in Fig 22, the results indicate that the communication delay of our protocol is lower than that of Ref. [25,51] but slightly higher than that of Ref. [89]. Regarding total end-to-end latency, our method results in greater latency than the others; however, this increase is deemed acceptable given the enhanced security features achieved.

**Table 6:** Comparison of computation overhead between the proposed scheme and the methods in refs. [25,89].

Scheme	Computation delay (ms)	Communication delay (ms)	Total delay (ms)
Proposed scheme	5642.1141	3.85	5645.9
Method of [25] (2021)	39.3104	4.017	43.32
Method of [89] (2021)	453.795	1.3696	455.165
Method of [51] (2024)	1222.9	4.488	1227.4

#### Energy consumption.

**Fig 23:**
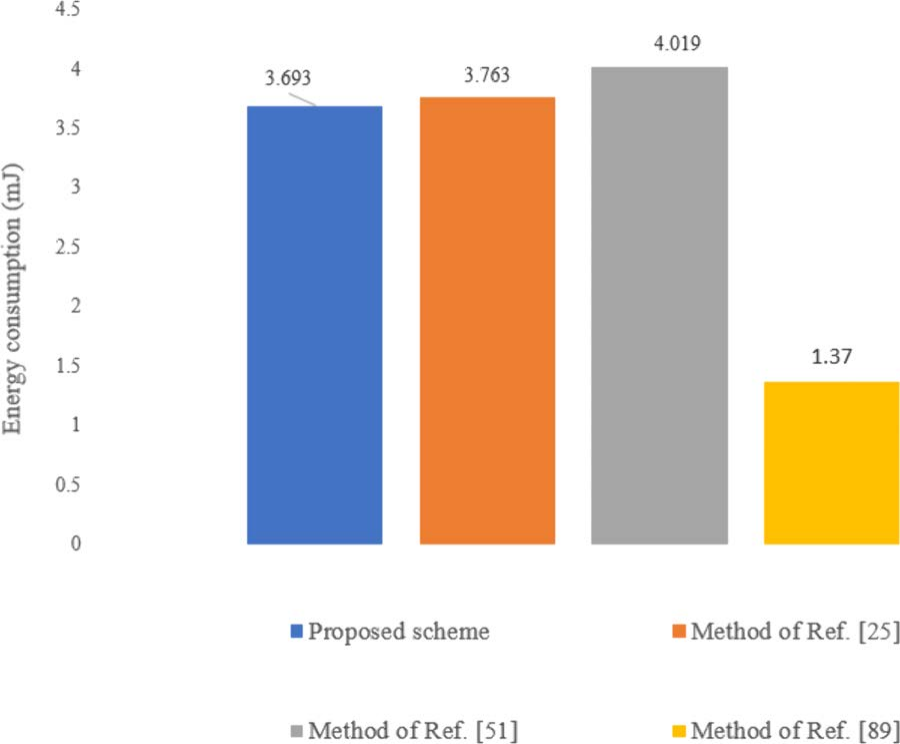
Sensor energy consumption of the proposed scheme compared to the methods in Refs. [25,51,89].

Given that sensors have limited energy resources and their energy consumption is a critical factor, we will focus solely on the energy usage of the sensors. The energy consumption for a MICA2 sensor is calculated using the formula E = U × I × t [94], where the values for current (I) and voltage (U) are assumed to be 8 mA and 3.0 Volts, respectively. The time (t) is derived from the execution times of operations listed in Table 2. Additionally, the energy consumption for receiving and transmitting a bit on the MICA2 sensor is 2.36 μJ/bit and 4.28 μJ/bit, respectively [98]. Table 7 presents the energy consumption for sensors using our proposed method compared to the methods in Refs. [25,51,89]. As shown in Fig 23, the energy consumption for sensors in our scheme is less than Refs. [25,51], while Ref. [89] results in lower energy consumption. However, the approach in Ref. [89] assumes a secure channel between the sensors and the patient's cellphone, focusing only on the energy needed to transmit raw data to the cellphone. The energy required to establish and maintain a secure channel between the sensors and the gateway is not accounted for, as its specifics are not provided. If this energy were included, the sensor's energy consumption in Ref. [89] could be higher.

**Table 7:** Comparison of sensor energy consumption in the proposed scheme with the methods in refs. [25,89].

Scheme	Energy consumption (mJ)		
	Communications	Computations	Total
Proposed scheme	3.097	0.596	3.693
[25] (2021)	3.23	0.533	3.763
[89] (2021)	1.37	–	1.37
[51] (2024)	3.399	0.620	4.019

### Security analysis

In this section, the security features of the proposed scheme are intuitively assessed. These security features include confidentiality, integrity, availability, privacy, anonymity, authentication, authorization, and scalability as discussed below:

- **Confidentiality**

Confidentiality ensures that only authorized users have access to sensitive data [62]. In step 3, critical information such as ${AmbiguousID}_{S}\text{ and }N_{y}$ are encrypted as ${BlindID}_{s}$ which are accessed only by end users. Moreover, in step 6, healthcare data (Info), the location of GW (position), and the identity of GW (${ID}_{GW}$) are encrypted as ${Blindposition}_{s}$. In steps 8 and 9, the UA should authenticate itself to access patients’ information. Using private BCs such as ${BC}_{sensor\_auth},$ ${BC}_{{Patient}_{j}},\;and$ $\overset{patients\_info}{\underset{i}{BC}}$, unauthorized access is prevented. In addition, in steps one, three, and five of Fig 3, the critical information is hashed as ${AID}_{s}$, $tr,\;{tr}_{N_{y}},V_{GW}$.

- **Integrity**

Data integrity refers to the assurance that data is received at its destination exactly as it was sent, without any alterations, and that it cannot be modified without authorization [38,62]. In step 6, healthcare information is encrypted as ${BlindPosition}_{s}$. Thanks to the immutability of blockchain technology, the integrity of this information is maintained. In step 7, the integrity of healthcare data is further secured by incorporating the hash of the data block for ${BC}_{{Patient}_{j}}$ and $\overset{patients\_info}{\underset{i}{BC}}$ into both $\overset{patients\_info}{\underset{i}{BC}}$ and ${BC}_{main}$, respectively. The sensor computes ${MAC}_{info}$ in step 5 to confirm the integrity of the healthcare data to the UA in step 9. Additionally, the AP calculates $V_{GW}$ to verify the integrity of ${T_{seq}}_{new}$ to the sensor.

- **Availability**

Patients and end users can access the healthcare system even if their previous AP is unavailable, as all APs can assist them by utilizing ${BC}_{sensor\_auth}.$

- **Privacy**

In the proposed method, unauthorized adversaries cannot access sensitive information or location data. The healthcare information is encrypted and stored in the local database of the APs, with its hash saved in ${BC}_{{Patient}_{j}}$ *to* prevent any modifications. Additionally, the AP records the hash of the data block from ${BC}_{{Patient}_{j}}$ in both $\overset{patients\_info}{\underset{i}{BC}}$ and ${BC}_{main}$. The GW authenticates itself to the AP using Idemix, which employs ZKP authentication, ensuring that the true identity of the GW supporting the user is not disclosed to the AP. Consequently, the user's location remains concealed even if the GW is known. Furthermore, the sensor utilizes a one-time token, and the AP encrypts the ambiguous identities of patients’ sensors, their locations, and healthcare data using the UA public key through ${BlindID}_{s}$ and ${BlindPosition}_{\text{s}}$, respectively.

- **Anonymity**

In this paper, the true identities of sensors and GWs are protected from both unauthorized entities and external attackers. By utilizing one-time tokens and shadow identities, the anonymity of the sensors is maintained against GWs and external threats. Patients store ${AmbiguousID}_{S}$, ${AmbiguousK}_{S},{\;verify}_{index}$ and HSID in the private blockchain*,* ${BC}_{sensor\_auth}$, which is only accessible to authorized APs. Both GWs and patients employ Idemix for anonymous authentication to the AP using the ZKP technique.

- **Authentication**

The sensor employs a one-time token and shadow identities, specifically ${AmbiguousID}_{S}$ and ${AmbiguousK}_{S}$, for anonymous authentication to the GWs with assistance from the APs. Additionally, the private blockchain (${BC}_{sensor\_auth}$) is utilized to store critical sensor information, such as ${AmbiguousID}_{S},$ ${AmbiguousK}_{S}$, ${verify}_{index}$, and HSID. GWs use Idemix for anonymous authentication to the APs, while UAs and APs rely on public keys and certificates for mutual authentication.

- **Authorization**

In this context, authorization refers to the fact that only designated UAs are permitted to access patients’ information. The ambiguous identities of patients’ sensors, their locations, and healthcare data are encrypted using the UA’s public key as ${BlindID}_{s}$ and ${BlindPosition}_{s}$ ensuring that only authorized users can access sensitive patient information.

- **Scalability**

Scalability refers to the ability to manage a growing volume of data and an increasing number of users within the system [99]. Our protocol achieves scalability by utilizing network clustering and storing only the hash of the healthcare data in ${BC}_{{Patient}_{j}}.$

Additionally, we address the security of the proposed scheme against various attacks, including Denial of Service (DoS), modification, mining, storage, replay, impersonation, man-in-the-middle attacks, and collusion, which are discussed in detail below.

- **Denial of Service (DoS)**

This attack occurs when numerous transactions are directed to a destination in an attempt to disrupt its availability [47]. By employing network clustering, we reduce both the impact and likelihood of a Denial of Service (DoS) attack. This means that if an attack does occur, only a portion of the nodes will be affected, rather than all of them. Moreover, using private BC (${BC}_{sensor\_auth}$) helps all application providers to serve the patients. Therefore, if one AP goes out of reach, the others can still serve its users. In addition, our scheme checks the UA authentication and has limitations in the number of acceptable unsuccessful access requests.

- **Modification**

Attackers may modify or delete patients’ data [47], which includes healthcare information, ${AmbiguousID}_{S},\;{AmbiguousK}_{S}$, ${verify}_{index},$ and HSID during a modification attack. In step 3, the patient's critical information, ${ID}_{s},\;$and $N_{y}$ are encrypted as ${BlindID}_{s}$. In step 6, the gateway's ID (${ID}_{GW}$), the location of the GW, and healthcare information are encrypted as ${Blindposition}_{s}$. Additionally, in step 7, healthcare data is encrypted and stored in the local database of the APs. The hash of the healthcare data is also saved in ${BC}_{{Patient}_{j}}$. Furthermore, the hash values of the data blocks from ${BC}_{{Patient}_{j}}$ and $\overset{patients\_info}{\underset{i}{BC}}$ are stored in $\overset{patients\_info}{\underset{i}{BC}}$ and ${BC}_{main}$, respectively. Finally, the end user can verify the integrity of the healthcare information through the message authentication code (${MAC}_{info}$) calculated by the sensor in step 5.

- **Mining**

In a mining attack, a group of nodes, constituting 51% of the system, collaborates to introduce a fraudulent chain into the blockchain [39]. To mitigate the risk of such an attack, our scheme employs a private blockchain (${BC}_{main}$*)* that stores the hash of the last block from each private blockchain in the overlay network (i.e., $\overset{patients\_info}{\underset{\text{1}}{BC}},\overset{patients\_info}{\underset{\text{2}}{\;BC}},\;\ldots \overset{patients\_info}{\underset{N}{,\;BC}}$)*.*

- **Storage**

A storage attack involves attackers deleting, modifying, or adding data within the database [62]. In step 7, the hash values of healthcare information are recorded in ${BC}_{{Patient}_{j}}$. Additionally, the hash values of the data blocks from ${BC}_{{Patient}_{j}}$ are stored in $\overset{patients\_info}{\underset{i}{BC}}$. Furthermore, the hash values of the data blocks from $\overset{patients\_info}{\underset{i}{BC}}$ are stored in ${BC}_{main}$ to help detect any modifications.

- **Replay**

In a replay attack, attackers resend intercepted messages from previous sessions to gain access to information without proper authentication [100]. Our scheme is resistant to replay attacks because it employs a one-time token and shadow identity in step 1, along with a timestamp in the calculation of ${Blindposition}_{s}$.

- **Impersonation**

In this attack, an attacker forges the identity of one of the real system entities and acts instead of that entity for malicious purposes. During sensor authentication, a one-time token, $T_{seq}$, and an identifier, ${AID}_{s}$, are used by sensors. Moreover, these two values and the shadow identifiers are only available to the sensors. Therefore, an attacker cannot impersonate the sensors. Besides, at the end of each session, the value of $T_{seq}$ is updated. Moreover, in step 2, the GW is anonymously authenticated to the AP using Idemix. Since the GWs utilize a zero-knowledge proof scheme, an attacker is not able to forge herself/himself as a trusted GW. In the case of APs, they utilize their private keys to sign a random variable and authenticate themselves to the GWs. As a result, the attacker is not successful to act as a valid AP. Similarly, for UA authentication to the APs, the UAs utilize their private keys. Therefore, attackers are not able to impersonate themselves as valid application users.

- **Man-in-the-Middle**

As previously mentioned, our scheme ensures mutual authentication between system entities, which helps prevent man-in-the-middle attacks, making our proposed solution resilient to this threat. Additionally, by incorporating a random variable, a one-time token, and an ambiguous key for sensor authentication, the protocol further defends against man-in-the-middle attacks. The gateway also authenticates itself anonymously as a valid gateway using Idemix. Furthermore, the use of private keys and timestamps in the authentication process for APs and UAs enhances the protocol's resistance to this type of attack. Consequently, it is not possible to use messages from previous sessions in the current session.

- **Collusion**

Since the real identity of patients and their sensors are not revealed to other entities, the collusion between them cannot expose the real identity of patients. Also, the AP only accesses to the ambiguous identities of sensors and encrypted healthcare information. Therefore, the proposed scheme is safe against the collusion of system entities.

As shown in Table 8, we compare the proposed method to previous researches (both the ones that use centralized infrastructure and those which exploit blockchain) in terms of security features and resistance against attacks. As seen, compared to related work, the proposed method provides more security features. Considering the selected centralized method presented in Ref. [25], it is obvious that the scheme faces a single point of failure problem. Therefore, in case of any problem for the network management (NM) entity, it is not possible to serve patients, and therefore the availability and scalability of the system are highly dependent on the availability of NM. Also, the data is sent without encryption, which endangers the confidentiality of the data.

In comparison to the most similar blockchain-based method presented in Ref. [89], it is important to note that their scheme primarily addresses privacy preservation for only a portion of the end-to-end communication path. They rely on trusted entities and assume a secure connection between the sensors and gateways. Additionally, they do not account for issues such as collusion, patient anonymity, and untraceability. In contrast, our proposed method leverages the distributed nature of blockchain and utilizes clustering to ensure availability and scalability, while also avoiding a single point of failure. We maintain end-to-end privacy and assume the presence of semi-honest entities. Our approach is resilient to collusion among internal entities, and patient anonymity is ensured through the anonymous authentication of sensors and gateways.

## Conclusion

This paper presents an end-to-end privacy-preserving protocol that utilizes Idemix with Hyperledger Fabric to safeguard patient privacy. The proposed scheme ensures the anonymity of patients against both internal and external threats. Idemix achieves identity privacy through a blind signature scheme and efficient zero-knowledge proofs. Given the resource constraints of sensors, it employs lightweight operations such as XOR and hashing for securing sensor communications. Importantly, the real identities of sensors and gateways (GWs) remain concealed from APs, preventing them from linking healthcare information, location data, and patient identities. This effectively protects both location privacy and data privacy from inquisitive APs. Additionally, patients’ identities are not exposed to the GWs, ensuring that privacy is maintained against these entities as well. Only authorized user applications (UAs) can access patients’ healthcare information and locations. Consequently, each intermediary entity possesses only a fragment of patient information, preventing them from connecting identity, location, and healthcare data. The GWs can access only location and healthcare information without knowing the patients’ true identities, while the APs can only see the obfuscated identities of patients and their sensors.

A comprehensive security analysis indicates that our protocol ensures confidentiality, integrity, availability, authentication, authorization, anonymity, and scalability, thus providing end-to-end privacy in healthcare systems. It further demonstrates resilience against various attacks, including DoS, modification, mining, storage, and replay attacks. In contrast to traditional centralized methods, our approach is also resistant to collusion attacks. Moving forward, we suggest implementing flexible privacy levels tailored to individual patient preferences as an extension of the proposed method.

## Supporting information

S1 File All generated data from analysis of compared protocols. (XLSX)

## References

[1] Moosavi SR, et al. End-to-end security scheme for mobility enabled healthcare Internet of Things. Future Generation Computer Systems. 2016;64:108–24.

[2] Gope P, Hwang T. BSN-Care: A Secure IoT-Based Modern Healthcare System Using Body Sensor Network. IEEE Sensors J. 2016;16(5):1368–76. doi: 10.1109/jsen.2015.2502401

[3] Porambage P, Ylianttila M, Schmitt C, Kumar P, Gurtov A, Vasilakos AV. The quest for privacy in the internet of things. IEEE Cloud Comput. 2016;3(2):36–45.

[4] Chiuchisan I, Geman O. An approach of a decision support and home monitoring system for patients with neurological disorders using internet of things concepts. WSEAS Transactions on Systems. 2014;13:460–9.

[5] Crosby GV, Ghosh T, Murimi R, Chin CA. Wireless Body Area Networks for Healthcare: A Survey. International Journal of Ad Hoc, Sensor & Ubiquitous Computing. 2012;3(3):1–26. doi: 10.5121/ijasuc.2012.3301

[6] Alemdar H, Ersoy C. Wireless sensor networks for healthcare: A survey. Computer Networks. 2010;54(15):2688–710. doi: 10.1016/j.comnet.2010.05.003

[7] Fernandes CO, de Lucena CJP. An Internet of Things Application with an Accessible Interface for Remote Monitoring Patients. In: International Conference of Design, User Experience, and Usability. 2015. p. 651–61.

[8] Prakash R, Girish SV, Ganesh AB. Real-time remote monitoring of human vital signs using internet of things (IoT) and GSM connectivity. In: Proceedings of the International Conference on Soft Computing Systems. 2016. p. 47–56.

[9] Zhao Z. An efficient anonymous authentication scheme for wireless body area networks using elliptic curve cryptosystem. J Med Syst. 2014;38(2):13. doi: 10.1007/s10916-014-0013-5 24481718

[10] Yeh K-H. BSNCare+: A Robust IoT-Oriented Healthcare System with Non-Repudiation Transactions. Applied Sciences. 2016;6(12):418. doi: 10.3390/app6120418

[11] Gope P, Hwang T. Untraceable Sensor Movement in Distributed IoT Infrastructure. IEEE Sensors J. 2015;15(9):5340–8. doi: 10.1109/jsen.2015.2441113

[12] Liu J, Zhang Z, Chen X, Kwak KS. Certificateless Remote Anonymous Authentication Schemes for WirelessBody Area Networks. IEEE Trans Parallel Distrib Syst. 2014;25(2):332–42. doi: 10.1109/tpds.2013.145

[13] Aazam M, Hung PP, Huh EN. Smart gateway based communication for cloud of things. In: Intelligent Sensors, Sensor Networks and Information Processing (ISSNIP), 2014 IEEE Ninth International Conference on. 2014. p. 1–6.

[14] Desai P, Sheth A, Anantharam P. Semantic gateway as a service architecture for iot interoperability. In: Mobile Services (MS), 2015 IEEE International Conference on. 2015. p. 313–9.

[15] Negash B, Rahmani AM, Liljeberg P, Jantsch A. Fog Computing Fundamentals in the Internet-of-Things. Fog Computing in the Internet of Things. Springer; 2018. p. 3–13.

[16] Shuai M, Liu B, Yu N, Xiong L, Wang C. Efficient and privacy-preserving authentication scheme for wireless body area networks. Journal of Information Security and Applications. 2020;52:102499. doi: 10.1016/j.jisa.2020.102499

[17] Soufiene BO, Bahattab AA, Trad A, Youssef H. RESDA: Robust and Efficient Secure Data Aggregation Scheme in Healthcare using the IoT. In: 2019 International Conference on Internet of Things, Embedded Systems and Communications (IINTEC). 2019. p. 209–13. doi: 10.1109/iintec48298.2019.9112125

[18] Saha R, Kumar G, Rai MK, Thomas R, Lim SJ. Privacy Ensured ${e} $-Healthcare for Fog-Enhanced IoT Based Applications. IEEE Access. 2019;7:44536–43.

[19] Deebak BD, Al-Turjman F, Aloqaily M, Alfandi O. An Authentic-Based Privacy Preservation Protocol for Smart e-Healthcare Systems in IoT. IEEE Access. 2019;7:135632–49. doi: 10.1109/access.2019.2941575

[20] Tang W, Ren J, Deng K, Zhang Y. Secure Data Aggregation of Lightweight E-Healthcare IoT Devices With Fair Incentives. IEEE Internet Things J. 2019;6(5):8714–26. doi: 10.1109/jiot.2019.2923261

[21] Jain SK, Kesswani N. IoTP an Efficient Privacy Preserving Scheme for Internet of Things Environment. International Journal of Information Security and Privacy. 2020;14(2):116–42. doi: 10.4018/ijisp.2020040107

[22] Li S, Zhao S, Min G, Qi L, Liu G. Lightweight Privacy-Preserving Scheme Using Homomorphic Encryption in Industrial Internet of Things. IEEE Internet Things J. 2022;9(16):14542–50. doi: 10.1109/jiot.2021.3066427

[23] Rana S, Mishra D, Arora R. Privacy-Preserving Key Agreement Protocol for Fog Computing Supported Internet of Things Environment. Wireless Pers Commun. 2021;119(1):727–47. doi: 10.1007/s11277-021-08234-4

[24] Baek S, Seo S-H, Kim S. Preserving Patient’s Anonymity for Mobile Healthcare System in IoT Environment. International Journal of Distributed Sensor Networks. 2016;12(7):2171642. doi: 10.1177/155014772171642

[25] Nasr Esfahani M, Shahgholi Ghahfarokhi B, Etemadi Borujeni S. End-to-end privacy preserving scheme for IoT-based healthcare systems. Wireless Netw. 2021;27(6):4009–37. doi: 10.1007/s11276-021-02652-9 PMC13028556 41894424

[26] Sharavanan PT, Sridharan D, Kumar R. A Privacy Preservation Secure Cross Layer Protocol Design for IoT Based Wireless Body Area Networks Using ECDSA Framework. J Med Syst. 2018;42(10):196. doi: 10.1007/s10916-018-1050-2 30215143

[27] Babu MSS, Balasubadra K. Chronic privacy protection from source to sink in sensor network routing. International Journal of Applied Engineering Research. 2018;13(5):2798–808.

[28] Yeh K-H. A Secure IoT-Based Healthcare System With Body Sensor Networks. IEEE Access. 2016;4:10288–99. doi: 10.1109/access.2016.2638038

[29] Azaria A, Ekblaw A, Vieira T, Lippman A. Medrec: Using blockchain for medical data access and permission management. In: 2016 2nd International Conference on Open and Big Data (OBD). 2016. p. 25–30.

[30] Xia Q, Sifah EB, Asamoah KO, Gao J, Du X, Guizani M. MeDShare: Trust-Less Medical Data Sharing Among Cloud Service Providers via Blockchain. IEEE Access. 2017;5:14757–67. doi: 10.1109/access.2017.2730843

[31] Roehrs A, da Costa CA, da Rosa Righi R. OmniPHR: A distributed architecture model to integrate personal health records. Journal of Biomedical Informatics. 2017;71:70–81. 28545835 10.1016/j.jbi.2017.05.012

[32] Zhang J, Xue N, Huang X. A Secure System For Pervasive Social Network-Based Healthcare. IEEE Access. 2016;4:9239–50. doi: 10.1109/access.2016.2645904

[33] Yue X, Wang H, Jin D, Li M, Jiang W. Healthcare Data Gateways: Found Healthcare Intelligence on Blockchain with Novel Privacy Risk Control. J Med Syst. 2016;40(10):218. doi: 10.1007/s10916-016-0574-6 27565509

[34] Ekblaw A, Azaria A, Halamka JD, Lippman A. A case study for blockchain in healthcare: “MedRec” prototype for electronic health records and medical research data. In: Proceedings of IEEE open & big data conference. 2016. p. 13.

[35] Linn LA, Koo MB. Blockchain for health data and its potential use in health it and health care related research. In ONC/NIST Use of Blockchain for Healthcare and Research Workshop. Gaithersburg, Maryland, United States: ONC/NIST; 2016. p. 1–10.

[36] Ivan D. Moving toward a blockchain-based method for the secure storage of patient records. In ONC/NIST Use of Blockchain for Healthcare and Research Workshop. Gaithersburg, Maryland, United States: ONC/NIST; 2016. p. 1–11.

[37] Brodersen C, et al. Blockchain: Securing a new health interoperability experience. Accenture LLP. 2016.

[38] Hossein KM, Esmaeili ME, Dargahi T, khonsari A. Blockchain-based privacy-preserving healthcare architecture. In 2019 IEEE Canadian Conference of Electrical and Computer Engineering (CCECE). IEEE; 2019. p. 1–4.

[39] Dagher GG, Mohler J, Milojkovic M, Marella PB. Ancile: Privacy-preserving framework for access control and interoperability of electronic health records using blockchain technology. Sustainable Cities and Society. 2018;39:283–97. doi: 10.1016/j.scs.2018.02.014

[40] Guo R, Shi H, Zhao Q, Zheng D. Secure Attribute-Based Signature Scheme With Multiple Authorities for Blockchain in Electronic Health Records Systems. IEEE Access. 2018;6:11676–86. doi: 10.1109/access.2018.2801266

[41] Al Omar A, Bhuiyan MZA, Basu A, Kiyomoto S, Rahman MS. Privacy-friendly platform for healthcare data in cloud based on blockchain environment. Future Generation Computer Systems. 2019;95:511–21.

[42] Wang H, Song Y. Secure Cloud-Based EHR System Using Attribute-Based Cryptosystem and Blockchain. J Med Syst. 2018;42(8):152. doi: 10.1007/s10916-018-0994-6 29974270

[43] Sun Y, Zhang R, Wang X, Gao K, Liu L. A decentralizing attribute-based signature for healthcare blockchain. In 2018 27th International conference on computer communication and networks (ICCCN). IEEE; 2018. p. 1–9.

[44] Lee CH, Kim K-H. Implementation of IoT system using block chain with authentication and data protection. In 2018 International Conference on Information Networking (ICOIN). IEEE; 2018. p. 936–40

[45] Rahulamathavan Y, Phan RC-W, Rajarajan M, Misra S, Kondoz A. Privacy-preserving blockchain based IoT ecosystem using attribute-based encryption. In 2017 IEEE International Conference on Advanced Networks and Telecommunications Systems (ANTS). IEEE; 2017. p. 1–.

[46] Uddin MA, Stranieri A, Gondal I, Balasubramanian V. A patient agent to manage blockchains for remote patient monitoring. Studies in Health Technology and Informatics. 2018;254:105–15. 30306963

[47] Dorri A, Kanhere SS, Jurdak R. Towards an optimized blockchain for IoT. In 2017 IEEE/ACM Second International Conference on Internet-of-Things Design and Implementation (IoTDI). IEEE; 2017. p. 173–178

[48] Uddin MdA, Stranieri A, Gondal I, Balasubramanian V. Blockchain leveraged decentralized IoT eHealth framework. Internet of Things. 2020;9:100159. doi: 10.1016/j.iot.2020.100159

[49] Uddin MdA, Stranieri A, Gondal I, Balasubramanian V. Continuous Patient Monitoring With a Patient Centric Agent: A Block Architecture. IEEE Access. 2018;6:32700–26. doi: 10.1109/access.2018.2846779

[50] Gordon WJ, Catalini C. Blockchain technology for healthcare: facilitating the transition to patient-driven interoperability. Computational and Structural Biotechnology Journal. 2018;16:224–30. 30069284 10.1016/j.csbj.2018.06.003 PMC6068317

[51] Nasr Esfahani M, Ghahfarokhi BS, Etemadi Borujeni S. Blockchain-based end-to-end privacy-preserving scheme for IoT-based healthcare systems. J Supercomput. 2023;80(2):2067–127. doi: 10.1007/s11227-023-05522-7

[52] Farouk A, Alahmadi A, Ghose S, Mashatan A. Blockchain platform for industrial healthcare: Vision and future opportunities. Computer Communications. 2020;154:223–35.

[53] Simić M, Sladić G, Milosavljević B. A case study IoT and blockchain powered healthcare. Proc ICET. 2017:1–4.

[54] Brogan J, Baskaran I, Ramachandran N. Authenticating health activity data using distributed ledger technologies. Computational and Structural Biotechnology Journal. 2018;16:257–66. 30101004 10.1016/j.csbj.2018.06.004 PMC6071583

[55] Tripathi G, Ahad MA, Paiva S. S2HS- A blockchain based approach for smart healthcare system. Healthc (Amst). 2020;8(1):100391. doi: 10.1016/j.hjdsi.2019.100391 31753750

[56] Tanwar S, Parekh K, Evans R. Blockchain-based electronic healthcare record system for healthcare 4.0 applications. Journal of Information Security and Applications. 2020;50:102407. doi: 10.1016/j.jisa.2019.102407

[57] Hassan MU, Rehmani MH, Chen J. Privacy preservation in blockchain based IoT systems: Integration issues, prospects, challenges, and future research directions. Future Generation Computer Systems. 2019;97:512–29.

[58] Xu J, Xue K, Li S, Tian H, Hong J, Hong P, et al. Healthchain: A Blockchain-Based Privacy Preserving Scheme for Large-Scale Health Data. IEEE Internet Things J. 2019;6(5):8770–81. doi: 10.1109/jiot.2019.2923525

[59] Fu J, Wang N, Cai Y. Privacy-Preserving in Healthcare Blockchain Systems Based on Lightweight Message Sharing. Sensors. 2020;20(7):1898. 32235389 10.3390/s20071898 PMC7180853

[60] Wang DrH. IoT based Clinical Sensor Data Management and Transfer using Blockchain Technology. JISMAC. 2020;2(3):154–9. doi: 10.36548/jismac.2020.3.003

[61] Wang J, Han K, Alexandridis A, Chen Z, Zilic Z, Pang Y, et al. A blockchain-based eHealthcare system interoperating with WBANs. Future Generation Computer Systems. 2020;110:675–85. doi: 10.1016/j.future.2019.09.049

[62] Dwivedi AD, Srivastava G, Dhar S, Singh R. A Decentralized Privacy-Preserving Healthcare Blockchain for IoT. Sensors (Basel). 2019;19(2):326. doi: 10.3390/s19020326 30650612 PMC6359727

[63] McGhin T, Choo K-KR, Liu CZ, He D. Blockchain in healthcare applications: Research challenges and opportunities. Journal of Network and Computer Applications. 2019;135:62–75. doi: 10.1016/j.jnca.2019.02.027

[64] Stamatellis C, Papadopoulos P, Pitropakis N, Katsikas S, Buchanan WJ. A Privacy-Preserving Healthcare Framework Using Hyperledger Fabric. Sensors (Basel). 2020;20(22):6587. doi: 10.3390/s20226587 33218022 PMC7698751

[65] Sabiri K, Sousa F, Rocha T. A systematic review of privacy-preserving blockchain applications in healthcare. Multimed Tools Appl. 2025;84(32):39925–80. doi: 10.1007/s11042-024-20541-z

[66] Hasnain M, Albogamy FR, Alamri SS, Ghani I, Mehboob B. The Hyperledger fabric as a blockchain framework preserves the security of electronic health records. Frontiers in Public Health. 2023;11:1272787. 38089022 10.3389/fpubh.2023.1272787 PMC10713743

[67] Enare Abang J, Takruri H, Al-Zaidi R, Al-Khalidi M. Latency performance modelling in hyperledger fabric blockchain: Challenges and directions with an IoT perspective. Internet of Things. 2024;26:101217. doi: 10.1016/j.iot.2024.101217

[68] Juyal S, Sharma S, Harbola A, Shukla AS. Privacy and Security of IoT based Skin Monitoring System using Blockchain Approach. In 2020 IEEE International Conference on Electronics, Computing and Communication Technologies (CONECCT). IEEE; 2020. p. 1–5.

[69] Noether S, Mackenzie A, Research Lab TM. Ring Confidential Transactions. Ledger. 2016;1:1–18. doi: 10.5195/ledger.2016.34

[70] Axon L, Goldsmith M. PB-PKI: A privacy-aware blockchain-based PKI. 14th International Conference on Security and Cryptography (SECRYPT 2017). vol. 6, SCITEPRESS, 2016

[71] Tschorsch F, Scheuermann B. Bitcoin and Beyond: A Technical Survey on Decentralized Digital Currencies. IEEE Commun Surv Tutorials. 2016;18(3):2084–123. doi: 10.1109/comst.2016.2535718

[72] Moin S, Karim A, Safdar Z, Safdar K, Ahmed E, Imran M. Securing IoTs in distributed blockchain: Analysis, requirements and open issues. Future Generation Computer Systems. 2019;100:325–43.

[73] Wang D, Zhao J, Mu C. Research on Blockchain-Based E-Bidding System. Applied Sciences. 2021;11(9):4011. doi: 10.3390/app11094011

[74] Gu J, Sun B, Du X, Wang J, Zhuang Y, Wang Z. Consortium Blockchain-Based Malware Detection in Mobile Devices. IEEE Access. 2018;6:12118–28. doi: 10.1109/access.2018.2805783

[75] Blockchain Network. 2022. [Online]. [Accessed: Jul. 23, 2022]. Available: https://hyperledger-fabric.readthedocs.io/en/release-2.2/network/network.html

[76] Kyazhin S, Popov V. Yet Another E-Voting Scheme Implemented Using Hyperledger Fabric Blockchain. In International Conference on Computational Science and Its Applications. Springer; 2020. p. 37–47.

[77] Ledger. 2022. [Online]. [Accessed: Jul. 23, 2022]. Available:https://hyperledger-fabric.readthedocs.io/en/release-2.2/ledger/ledger.html

[78] Smart Contracts and Chaincode. 2022. [Online]. [Accessed: Jul. 23, 2022]. Available:https://hyperledger-fabric.readthedocs.io/en/release-2.2/smartcontract/smartcontract.html

[79] Peers. 2020-2022. [Online]. Available: https://hyperledger-fabric.readthedocs.io/en/latest/peers/peers.html

[80] MSP Implementation with Identity Mixer. 2020-2022. [Online]. Available: https://hyperledger-fabric.readthedocs.io/en/release-2.4/idemix.html

[81] Chaum D. Blind signatures for untraceable payments. Advances in cryptology. Springer; 1983. p. 199–203.

[82] Support for identity mixer credentials in Fabric CA. 2018. [Online]. Available: https://docs.google.com/document/d/1iqCHtX-bG3Ey7bZmm9_b-oqu30jtlDJO9HhccVtekEE/edit

[83] hyperledger-fabric-ca Documentation. 8 Jul 2022. [Online]. Available: https://readthedocs.org/projects/hyperledger-fabric-ca/downloads/pdf/latest/

[84] Chaum DL. Untraceable electronic mail, return addresses, and digital pseudonyms. Commun ACM. 1981;24(2):84–90. doi: 10.1145/358549.358563

[85] Lysyanskaya A, Rivest RL, Sahai A, Wolf S. Pseudonym systems. In International Workshop on Selected Areas in Cryptography. Springer; 1999. p. 184–99.

[86] Camenisch J, Lysyanskaya A. Signature schemes and anonymous credentials from bilinear maps. In Annual international cryptology conference. Springer; 2004. p. 56–72.

[87] Au MH, Susilo W, Mu Y. Constant-size dynamic k-TAA. In International conference on security and cryptography for networks. Springer; 2006. p. 111–25.

[88] Camenisch J, Dubovitskaya M, Lehmann A, Neven G, Paquin C, Preiss F-S. Concepts and languages for privacy-preserving attribute-based authentication. In IFIP Working Conference on Policies and Research in Identity Management. Springer: 2013. p. 34–52.

[89] Mohammad Hossein K, Esmaeili ME, Dargahi T, Khonsari A, Conti M. BCHealth: A Novel Blockchain-based Privacy-Preserving Architecture for IoT Healthcare Applications. Computer Communications. 2021;180:31–47. doi: 10.1016/j.comcom.2021.08.011

[90] Lincoln NK. Hyperledger Fabric 1.4.0 Performance Information Report. Available in https://hyperledger.github.io/caliper-benchmarks/fabric/resources/pdf/Fabric_1.4.0_javascript_node.pdf

[91] Sene I, Ciss AA, Niang O. I2PA, U-prove, and Idemix: An Evaluation of Memory Usage and Computing Time Efficiency in an IoT Context. In International Conference on e-Infrastructure and e-Services for Developing Countries. Springer; 2020. p. 140–53.

[92] Abbasinezhad-Mood D, Nikooghadam M. Efficient Design of a Novel ECC-Based Public Key Scheme for Medical Data Protection by Utilization of NanoPi Fire. IEEE Trans Rel. 2018;67(3):1328–39. doi: 10.1109/tr.2018.2850966

[93] Mahalle PN, Anggorojati B, Prasad NR, Prasad R. Identity Authentication and Capability Based Access Control (IACAC) for the Internet of Things. JCSANDM. 2013. doi: 10.13052/jcsm2245-1439.142

[94] Le XH, Lee S, Butun I, Khalid M, Sankar R, Kim M, et al. An energy-efficient access control scheme for wireless sensor networks based on elliptic curve cryptography. J Commun Netw. 2009;11(6):599–606. doi: 10.1109/jcn.2009.6388413

[95] Lai DTH, Palaniswami M, Begg R. Healthcare sensor networks: challenges toward practical implementation. CRC Press; 2011.

[96] Chatterjee S, Das AK. An effective ECC‐based user access control scheme with attribute‐based encryption for wireless sensor networks. Security and Communication Networks. 2015;8(9):1752–71.

[97] Wander AS, Gura N, Eberle H. Energy Analysis of Public--key Cryptography on Small Wireless Devices. In Proceedings of the 3rd IEEE Intl Conference on Pervasive Computing and Communications. California: IEEE Computer Society Press; 2005. p. 324–328.

[98] Calle M, Kabara J. Measuring energy consumption in wireless sensor networks using GSP. In 2006 IEEE 17th International Symposium on Personal, Indoor and Mobile Radio Communications. IEEE; 2006. p. 1–5.

[99] Yang J, Onik MMH, Lee N-Y, Ahmed M, Kim C-S. Proof-of-Familiarity: A Privacy-Preserved Blockchain Scheme for Collaborative Medical Decision-Making. Applied Sciences. 2019;9(7):1370. doi: 10.3390/app9071370

[100] Shen B, Guo J, Yang Y. MedChain: Efficient Healthcare Data Sharing via Blockchain. Applied Sciences. 2019;9(6):1207. doi: 10.3390/app9061207

[101] Luong DA, Park JH. Privacy-Preserving Blockchain-Based Healthcare System for IoT Devices Using zk-SNARK. IEEE Access. 2022;10:55739–52. doi: 10.1109/access.2022.3177211

[102] Sharma P, Namasudra S, Chilamkurti N, Kim B-G, Gonzalez Crespo R. Blockchain-Based Privacy Preservation for IoT-Enabled Healthcare System. ACM Trans Sen Netw. 2023;19(3):1–17. doi: 10.1145/3577926

